# The Highly Repeat-Diverse (Peri) Centromeres of White Lupin (*Lupinus albus* L.)

**DOI:** 10.3389/fpls.2022.862079

**Published:** 2022-04-05

**Authors:** André Marques, Bárbara Hufnagel, Alexandre Soriano, Benjamin Péret

**Affiliations:** BPMP, Institut Agro, CNRS, INRAE, Univ. Montpellier, Montpellier, France

**Keywords:** heterochromatin, repetitive DNA, centromere, CENH3, satellite DNA, cytogenomics

## Abstract

Plant genomes are known to be mainly composed of repetitive DNA sequences. Regardless of the non-genic function of these sequences, they are important for chromosome structure and stability during cell-cycle. Based on the recent available whole-genome assembly of white lupin (*Lupinus albus* L.; WL), we have *in silico* annotated and *in situ* mapped the main classes of DNA repeats identified with RepeatExplorer. A highly diverse and an abundance of satellite DNAs were found representing more than 10 families, where three of them were highly associated with CENH3-immunoprecipitated chromatin. Applying a strategy of several re-hybridization steps with different combinations of satDNA, rDNA, and LTR-RTs probes, we were able to construct a repeat-based chromosome map for the identification of most chromosome pairs. Two families of LTR retrotransposons, Ty1/copia SIRE and Ty3/gypsy Tekay, were highly abundant at pericentromeric regions, while the centromeric retrotransposon of WL (CRWL) from the CRM clade showed strong centromere-specific localization in most chromosomes and was also highly enriched with CENH3-immunoprecipitated chromatin. FISH mapping of repeat DNA showed some incongruences with the reference genome, which can be further used for improving the current version of the genome. Our results demonstrate that despite the relatively small genome of WL, a high diversity of pericentromeric repeats was found, emphasizing the rapid evolution of repeat sequences in plant genomes.

## Introduction

The genus *Lupinus* L., commonly known as lupins, is a genus of flowering plants in the legume family Leguminosae, comprising more than 300 species (Hughes and Eastwood, 2006; Drummond et al., 2012). Lupins are commonly known as beautiful ornamental plants, bearing numerous colorful flowers. They are grouped into Old world lupins (Mediterranean) and New world lupins (American) with a remarkable array of ecological habitats, justifying their interest as a case study for genome evolution, adaptation, and speciation (Ainouche and Bayer, 1999; Hughes and Eastwood, 2006). Among them, white lupin (*Lupinus albus* L.; WL) is distinct within the vast and polymorphous genus *Lupinus* because of a small variation of morphological characters with its origin attributed to the Mediterranean region. It is recognized as traditional food and feed of interest due to its high seed protein content (Bähr et al., 2014). However, it has wide intraspecific variability in physiological plant properties, likely due to its ancient cultivation, which started around 4,000 years ago (Gladstones, 1998).

The genus *Lupinus* is comprised of agronomic species of interest; however, only two species (*L. angustifolius* and *L. albus*) have fully sequenced genomes (Hane et al., 2017; Hufnagel et al., 2020). Despite its socioeconomic importance, the lack of genetic resources for WL has been hampering comprehensive genetic characterization. Recently, we have made available the first chromosome-level reference genome for WL (Hufnagel et al., 2020). This study was the first step in an effort to understand its main features, e.g., cluster root formation and seed quality properties, and support its future breeding. The availability of the high-quality genome assembly and annotation allowed us to understand how domestication of WL has impacted major traits such as seed quality and root developmental plasticity. Furthermore, the whole genome sequence allowed the identification and characterization of structural features, for instance, its repetitive sequence abundance, distribution, and characterization (Hufnagel et al., 2020).

The WL genome is highly repetitive and shows a great diversity of centromere-associated repeats. ChIPseq data showed that CENH3-containing chromatin is highly associated with the centromeric retrotransposon of WL (CRWL) and with at least four centromeric satellite DNA (cenDNA) repeats: CL2-5bp, CL10-78bp, CL21-38bp, and CL55-8bp. In contrast, the most abundant satellite repeat CL1-170bp did not show significant enrichment with the immunoprecipitated DNA. The CENH3-immunoprecipitated chromatin accounts for around 11% of the WL genome (Hufnagel et al., 2020). Along with these findings, a few more studies have been performed with respect to the characterization of other lupin chromosomes (Kaczmarek et al., 2009; Lesniewska et al., 2011; Ksiazkiewicz et al., 2013, 2015; Susek et al., 2016, 2017). Furthermore, we lack a comprehensive characterization of repeats across lupin chromosomes.

Here, we describe a high-quality chromosome mapping for the main repeats found in the genome of *L. albus* cv. AMIGA (2*n* = 50) (Hufnagel et al., 2020). We further show that the composition of its highly diverse (peri) centromeric heterochromatin allows, in most cases, the identification of chromosome-specific patterns, providing a centromere “bar code” for WL chromosomes. Our results demonstrate that despite the relatively small WL genome, the high diversity of pericentromeric repeats found provides yet another example of the rapid evolution of repeat sequences in plant genomes.

## Results

### Repeat Composition of WL (Peri) Centromeres

The repeat abundance and characterization of the WL genome were previously reported (Hufnagel et al., 2020). Briefly, 60% of the WL genome is composed of repetitive sequences, with over 3/4 of its repeats (43.3% of the genome) matching known transposable elements (TEs). Typical of most plants, TEs were the most common long terminal repeats (LTRs) retrotransposons (34%), with a remarkable accumulation of Ty3/gypsy Tekay and CRM chromoviruses and Ty1/copia SIRE (16.6, 3.4, and 6.2%, respectively). Class II TEs accounted for ∼0.8% of the genome (Supplementary Table 1), according to Hufnagel et al. (2020).

**FIGURE 1:**
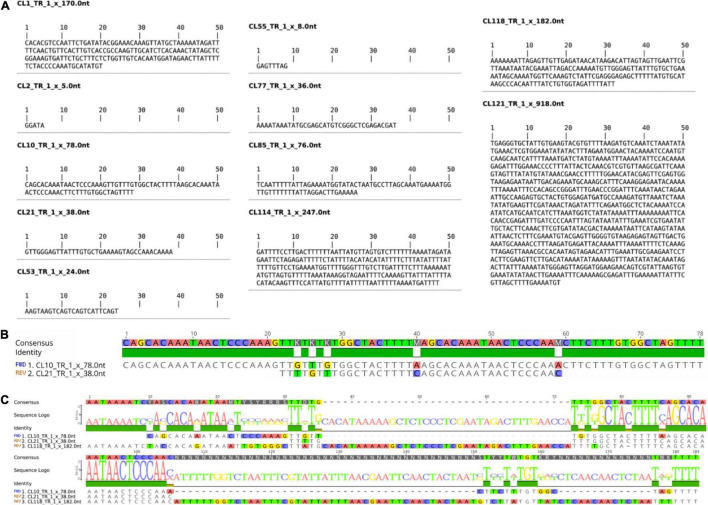
Consensus Monomer and Characterization of WL satDNA Repeats. **(A)** Consensus monomer sequences of 11 satDNA clusters identified. **(B)** Characterization of the CL10 and CL21 satDNA repeat family. **(C)** Comparison of CL10 and CL21 with CL118, which also belongs to the same satDNA family but has evolved a longer repeat unit.

Tandem repeats comprised ∼15% of the WL genome, distributed in 14 tandem repeat clusters (CL1, CL2, CL10, CL21, CL52, CL53, CL55, CL68, CL77, CL85, CL114, CL118, CL121, CL127 (Table 1). These tandem repeats varied from short monomer length with 5 bp consensus sequence (CL2) to very long monomers up to 918 bp (CL121) (Figure 1 and Supplementary Figure 1). After further characterization of the consensus sequence of the identified tandem repeats, we observed that CL52 and CL127 are very similar to CL1 and were grouped within a single supercluster, thus representing a subset of the same repeat comprising the most abundant tandem repeat family of WL. CL10 (78b p) and CL21 (38 bp) are similar satellites comprising different subfamilies. CL10 represents a dimer organization of CL21 with some sequence divergence (Figure 1B). CL118 (182 bp) also shared sequence similarity to CL10 and CL21 (Figure 1C), but at a lower level, indicating an intraspecific evolution of this satDNA family. Among the identified tandem repeats, CL1–CL52–CL127 (170 bp) and CL2 (5 bp) were highly abundant, together comprising ∼12% of the genome (Table 1). Further characterization of satellite sequences is provided in Supplementary Figure 1.

**TABLE 1:** Satellite DNA diversity in white lupin: genomic abundance, monomer length, and features.

SatDNA clusters	Genomic abundance (%)	Monomer length (bp)	Features*
CL1, CL52, CL127	6.129	170	Most abundant satDNA; No enrichment with CENH3-immunoprecipitated DNA
CL2	5.473	5	Mid-level enrichment with CENH3-immunoprecipitated DNA
CL10	1.290	78	High enrichment with CENH3-immunoprecipitated DNA
CL21	0.766	38	High enrichment with CENH3-immunoprecipitated DNA; specific to three chromosome pairs
CL53	0.303	24	No enrichment with CENH3-immunoprecipitated DNA
CL55	0.285	8	High enrichment with CENH3-immunoprecipitated DNA; specific to three chromosome pairs
CL77	0.117	36	No enrichment with CENH3-immunoprecipitated DNA
CL85	0.085	76	No enrichment with CENH3-immunoprecipitated DNA
CL114	0.027	247	No enrichment with CENH3-immunoprecipitated DNA
CL118	0.024	182	Similarity to CL10 and CL21; No enrichment with CENH3-immunoprecipitated DNA
CL121	0.023	918	LalbChr17-specific satDNA; No enrichment with CENH3-immunoprecipitated DNA
Total	14.713		

*CENH3-ChIPseq data from Hufnagel et al. (2020).

**FIGURE 2:**
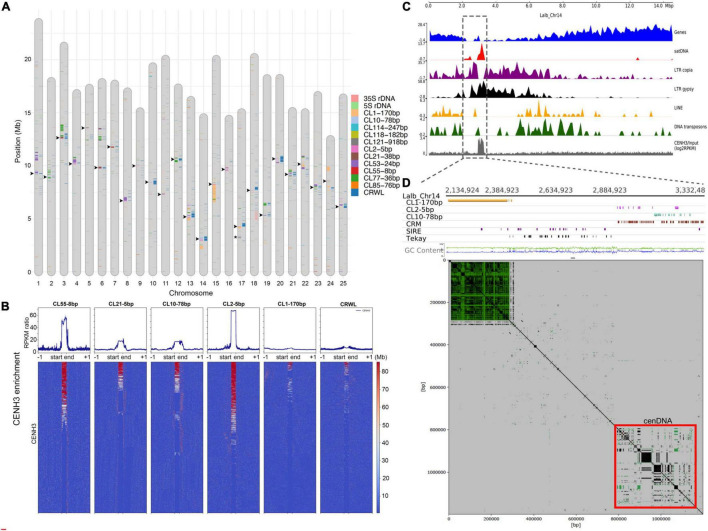
Repeat Composition and Characterization of WL Centromeres. **(A)** SatDNA, rDNA, and CRWL annotation of the reference genome of WL (*L. albus* cv. AMIGA). Arrowheads indicate the location of centromeres based on the CENH3-ChIPseq analysis. Asterisks indicate the chromosome-specific CL121-918bp satDNA array on Lalb-chr-17. **(B)** CENH3-ChIPseq enrichment profiles (RPKM ratio) for the centromeric satDNA families, CL1-170bp, and CRWL of WL. Note the specific high enrichment for CL2-5bp, CL10-78bp, CL21-38bp, and CL55-8bp. **(C)** Lalb_Chr14 as an example showing the enrichment for repeat sequences toward the proximal region. **(D)** A zoomed view of Lalb_Chr14 centromeric region, note that despite a large CL1-170bp array, the centromere function is specifically associated with a region enriched with CL2-5bp, CL10-78bp, and CRWL.

We performed full annotation of satDNA sequences and TEs at the chromosome-level in the WL genome. LTR retrotransposons were heavily concentrated at the pericentromere, in contrast to LINEs and Class II DNA transposons that were evenly distributed or in some cases more distally located in the assembled chromosomes (Figure 2 and Supplementary Figure 2). Chromosome-level annotation of satDNA sequences allowed us to observe that most WL pericentromeric regions are enriched with different satDNA families showing a chromosome-specific pattern (Figure 2A). Contrasting to the broader distribution of TEs, satDNA repeats were found to be more narrowly distributed (Figure 2 and Supplementary Figure 2).

Hufnagel et al. (2020) have shown that at least four satDNA families (CL2-5bp, CL10-78bp, CL21-38bp, and CL55-8bp) and the family of LTR/gypsy CRWL compose the functional centromeres of WL based on CENH3-ChIPseq experiments. However, the specific level of enrichment for CENH3 across all these repeats and the additional repeats found on WL were not studied in detail. Here, based on a detailed profiling and heatmap analysis of CENH3 ChIP enrichment for all repeat families, we have confirmed that these repeats are the only ones showing high association with functional centromeres (defined by CENH3 association). CL2-5bp satDNA showed the highest enrichment among all repeats, followed by CL55-8bp, CL10-78bp, and CL21-38bp, respectively. CRWL showed a higher enrichment only at the repeat start site (RSS) (Figures 2B,C). As an example, functional centromere in Chr14 is observed in association with an island of satDNA repeats and LTR gypsy elements at its proximal region (Figure 2C). A zoomed view of this chromosome further revealed that functional centromere is specifically associated with CL2-5bp and CL10-78bp satDNA repeats and the centromeric retrotransposon CRWL (Figure 2D). Remarkably, although CL1-170bp was by far the most abundant satDNA repeat, it did not show enrichment with CENH3 and seems to be mainly composing the pericentromeric heterochromatin in WL rather than occupying functional centromeres (Figures 2C,D). The position of functional centromeres was assigned to each chromosome on the WL reference genome based on the presence of these repeats (Figure 2A, arrowheads).

### Repeat-Based Fluorescence *in situ* Hybridization Mapping Allows the Assignment of WL Chromosomes

Given the abundance of class I TEs and the diversity of tandem repeats in WL, we aimed to assign the chromosomes based on the comparison of the *in silico* annotation of the WL reference genome and *in situ* hybridization patterns. Since TEs were not informative enough with a similar pattern of distribution in all chromosomes (Supplementary Figures 2, 3), we focused on the distribution of satDNA repeats by sequential multicolor FISH.

The highly diverse composition of WL pericentromeres observed *in silico* was also observed after sequential multicolor FISH and allowed us the identification of most chromosome pairs. Furthermore, we could potentially assign most of the assembled chromosomes of WL, whereas some contrasting patterns between the *in silico* annotation of the reference genome and *in situ* hybridization did not allow us to assign all chromosomes, indicating possible mis-assembled regions.

FISH signals were observed for CL1-170bp, CL2-5bp, CL10-78bp, CL21-38bp, CL53-24bp, CL55-8bp, CL68-telomere, CL77-36bp, CL121-918bp, as well as the 5S and 35S rDNA sequences. No obvious signals were observed for CL85-36bp, CL114-247bp, and CL118-182bp (not shown).

**FIGURE 3:**
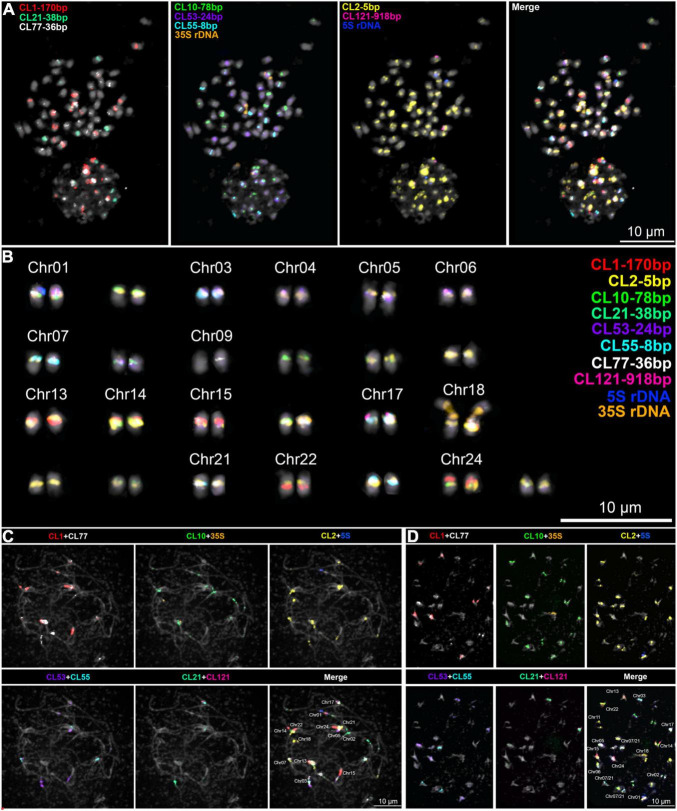
Sequential multicolor FISH mapping on **(A,B)** mitotic metaphase cell and meiotic **(C)** pachytene, and **(D)** diakinesis cells with the main satDNA repeats found on the WL genome. A karyogram of the cell in **(A)** is shown in **(B)**. A chromosome-specific pattern was observed and allowed the assignment of several chromosomes to the reference genome.

As shown previously, CRWL FISH signals were observed as narrow-distributed signals at the centromeric regions of most WL chromosomes (Supplementary Figure 4). Remarkably, a precise co-localization of signals of CRWL and the centromere-associated satDNA repeats (CL2-5bp, CL10-78bp, CL21-38bp, and CL55-8bp) was observed (Supplementary Figure 4). This association is also clearly observed at the sequence level as seen on the repeat annotation and do plots of WL centromeric regions (Supplementary Dataset 1). A wider distribution beyond the pericentromeres of WL chromosomes was observed for the LTR retrotransposon SIRE Ty1/Copia and Tekay Ty3/Gypsy (Supplementary Figures 3, 4). FISH signals of the CL1-170 bp repeat were observed in eight chromosome pairs, mostly seen as large blocks, apparently located in pericentromeric heterochromatin; CL10-78bp repeat-signals were observed in 14 chromosome pairs with a narrow distribution in centromeric regions; CL21-38bp and CL55-8bp repeat-signals were also observed as narrow centromeric signals in three different chromosome pairs, each serving as good markers for chromosome identification. Remarkably, CL10, CL21, and CL55 did not occur simultaneously in any chromosome. Furthermore, CL121-918 was found as a single FISH signal in a single chromosome pair LalbChr17. Also, 5S and 35S rDNA were found in a single chromosome pair each, LalbChr01 and LalbChr18, respectively, and served as good markers for chromosome identification and assignment. FISH mapping of the main repeats and the potentially assigned chromosomes are presented in Figure 3 and Supplementary Figures 4, 5, respectively. The potential assignment of WL chromosomes was based on the detailed comparison of the annotated pericentromeric regions of the reference genome (Figure 1 and Supplementary Dataset 1) and the pattern of FISH-signals observed (Figure 3 and Supplementary Figures 4, 5).

Below we describe the *in silico* and *in situ* observations of the main centromeric repeat distribution for all WL chromosomes:

**LalbChr01:** This chromosome is *in silico* characterized by harboring the single 5S rDNA array and having multiple CL53-24bp arrays. The putative centromere site sits on the flanking region of the first CL53-24bp array as depicted from the high accumulation of CRWL in this region (Figure 1 and Supplementary Dataset 1). Our FISH mapping also showed, in addition to 5S rDNA and CL53-24bp signals, strong signals for CL1-170bp, CL2-5bp, and CL10-78bp. The CL1-170bp signal was observed close to CL53-24bp signal, likely at the pericentromeric heterochromatin, while CL10-78bp and CL2-5bp signals colocalized with CRWL signal.

**LalbChr02:** This chromosome is *in silico* characterized by having large CL77-36bp and CL10-78bp arrays. This chromosome was not identified by *in situ* hybridization.

**LalbChr03:** This chromosome is both *in silico* and *in situ* characterized by having at the same time arrays for CL21-38bp (observed only in 3 chromosomes), CL2-5bp, and CL77-36bp.

**LalbChr04:** This chromosome is *in silico* characterized by having a large CL53-24bp array and additional CL2-5bp and CL77-36bp on opposite flanking sides. This chromosome was not identified by *in situ* hybridization.

**LalbChr05:** This chromosome is both *in silico* and *in situ* characterized by having the presence of CL1-170bp and CL21-38bp. Additionally *in situ* signals were observed for CL2-5bp.

**LalbChr06:** This chromosome is *in silico* characterized by having the presence of CL10-78bp, CL2-CL53-24bp, and CL77-36bp. However, *in situ* signals were observed only for CL55-8bp (present in three chromosomes) and a weak CL2-5bp.

**LalbChr07:** This chromosome is *in silico* characterized by having the presence of CL55-8bp and CL77-36bp. However, *in situ* signals were observed only for CL55-8bp (present in three chromosomes) and a weak CL2-5bp.

**LalbChr08:** This chromosome is both *in silico* and *in situ* characterized by having a large CL53-24bp satDNA block and additionally a minor CL2-5bp array.

**LalbChr09:** This chromosome is *in silico* characterized by a minor CL2-5bp array and low enrichment with CRWL. This chromosome was not identified by *in situ* hybridization.

**LalbChr10:** This chromosome is *in silico* characterized by minor CL2-5bp and CL10-78bp arrays, but highly enriched with CRWL in the flanking regions (Supplementary Dataset 1). This chromosome was not identified by *in situ* hybridization.

**LalbChr11:** This chromosome is *in silico* characterized by a minor CL2-5bp array and low enrichment with CRWL. This chromosome was not identified by *in situ* hybridization.

**LalbChr12:** This chromosome is *in silico* characterized by having two CL77-36bp arrays with a minor CL2-5bp array and enrichment of CRWL between them. This chromosome was not identified by *in situ* hybridization.

**LalbChr13:** This chromosome is both *in silico* and *in situ* characterized by having the largest CL1-170bp satDNA block and additionally CL2-5bp and CL53-24bp arrays.

**LalbChr14:** This chromosome is both *in silico* and *in situ* characterized by having a large CL1-170bp satDNA block and additionally CL2-5bp and CL10-78bp arrays.

**LalbChr15:** This chromosome is both *in silico* and *in situ* characterized by having the largest CL1-170bp satDNA block and additionally a minor CL2-5bp array. Although a CL53-24bp signal was observed on this chromosome (Figures 3A,B), no trace of this repeat was observed on the assembled chromosome.

**LalbChr16:** This chromosome is *in silico* characterized by having discrete arrays for CL1-170bp, CL2-5bp, and CL53-24bp. This chromosome was not identified by *in situ* hybridization.

**LalbChr17:** This chromosome is *in silico* characterized by having a single array for the satDNA CL121-918bp and an additional CL1-170bp satDNA array. Indeed, we could map this chromosome *in situ* because of the presence of CL121-918bp signal and also a signal for CL21-38bp, which allowed us to assign the third chromosome with a CL21-38bp cluster. Additionally, CL1-170bp and CL2-5bp hybridization signals were also observed.

**LalbChr18:** This chromosome is *in silico* characterized by having the 35S rDNA unit. *In situ* hybridization showed in addition to the 35S rDNA also a CL2-5bp signal.

**LalbChr19:** This chromosome is *in silico* characterized by having discrete arrays for CL1-170bp, CL10-78bp, CL53-24bp, and a larger array of CL77-36bp. This chromosome was not identified by *in situ* hybridization.

**LalbChr20:** This chromosome is *in silico* characterized by having a minor CL53-24bp satDNA and CL2-5bp arrays. This chromosome could not be identified by our repeat mapping *in situ* hybridization.

**LalbChr21:** This chromosome is *in silico* characterized by having the presence of CL2-5bp, CL10-78bp, CL55-8bp, and CL77-36bp. *In situ* signals were only observed for CL2-5bp, CL55-8bp, and CL77-36bp.

**LalbChr22:** This chromosome is *in silico* characterized by having the presence of a CL1-170bp large array and additional arrays for CL53-24bp and CL77-36bp. *In situ* signals were only clearly observed for CL1-170bp and occasionally for CL77-36bp.

**LalbChr23:** This chromosome is *in silico* characterized by two CL77-36bp arrays and a larger CL53-24bp array. This chromosome was not identified by *in situ* hybridization.

**LalbChr24:** This chromosome is *in silico* characterized by having the presence of a large CL1-170bp satDNA block and another additional minor array of the same satellite in the other chromosome arm. *In situ* signals were only observed for CL2-5bp, CL55-8bp, and CL77-36bp.

**LalbChr25:** This chromosome is *in silico* characterized by discrete arrays for CL1-170bp, CL2-5bp, and CL53-24bp. This chromosome was not identified by *in situ* hybridization.

The repeat annotation (TEs, satDNA, and rDNA) of the WL reference genome is available in the genome browser website,^1^ and the detailed GFF annotation files can be accessed and downloaded from there.

## Discussion

### WL Is Unique With Respect to Its Highly Diverse Pericentromeric Heterochromatin

*Lupinus* is a genus of considerable agronomic importance with a couple of species with full genome assemblies available (Hane et al., 2017; Hufnagel et al., 2020). Yet, detailed studies of the chromosomal characterization of repeats have been restricted to our recent study in WL (Hufnagel et al., 2020). Thus, the present study presents the first high-resolution characterization of the distribution of repetitive sequences for a lupin species. The heterochromatin in WL revealed it to be composed of many different tandem repeats with a rather intermingled organization pattern. Also, the availability of the full annotated genome assembly allowed us to perform a comparison with the observed chromosome distribution of the main repeats found in the WL genome. Remarkably, its heterochromatin seems to be mainly restricted to the pericentromeric regions in all chromosomes. Although some signatures of tandem repeats were found more at the terminal chromosomal regions in the annotated genome (see text footnote 1), this was not observed by FISH experiments (data not shown), which suggests a low abundance of these repeats.

Our FISH repeat mapping results showed some incongruences in the annotation of satDNA sequences in the WL reference genome (*L. albus* cv. AMIGA). These differences were mainly due to missing satDNA sequences at the highly repetitive (peri) centromeric regions in the reference genome. Tandem repeat-rich regions create a technical challenge to genome assembly (Treangen and Salzberg, 2011). For example, the *Arabidopsis* reference genome is one of the best-studied plant genome assemblies, but still contains many inaccuracies regarding satDNA array length and higher-order organization (Kim et al., 2014; Maheshwari et al., 2017). Only very recently, nearly complete telomere-to-telomere assemblies have been released for the genome of *A. thaliana* (Naish et al., 2021; Wang et al., 2021). For non-model organisms, assemblies often contain even less information, making the study of repetitive regions laborious and susceptible to failure.

In WL, all CENH3-enriched satDNA repeats CL2-5bp, CL10-78bp, CL21-38bp, and CL55-8bp were the most lacking expected regions in the reference genome compared to the FISH mapping results. For instance, CL2-5bp showed strong signals in most chromosomes but was found in the reference genome as short arrays in a few chromosomes. In addition, CL10-78bp showed FISH signals in eight chromosome pairs but was found in only five chromosomes in the reference genome. CL21-38bp and CL55-8bp showed signals in three distinct chromosome pairs each, but only two distinct chromosomes in the reference genome were found in each of these satDNAs. In the future, the use of more modern and robust sequence technology like highly accurate long PacBio HiFi reads (Wenger et al., 2019) may be important to improve the assembly of such regions in the reference genome of white lupin.

Most plant species normally show few satDNA repeats specifically associated with functional centromeres (Marques et al., 2015; Maheshwari et al., 2017) but WL seems to show a highly diverse functional centromere composition and provides a good example of centromere plasticity from the DNA point of view within a single species. However, highly diverse centromeric repeats can also be observed in other legume species like the case reported in *Pisum sativum*, where chromosomes possess several centromere domains per chromosome and are tightly associated with 13 distinct satDNA families and with one centromeric retrotransposon (CR) family (Neumann et al., 2012).

### Three Major satDNA Families Comprise Over 90% of satDNA Repeats in WL

As described in Hufnagel et al. (2020) and deeply depicted in this study, six different satDNA clusters were grouped within three major satDNA families. The most abundant satDNA, composed of CL1, CL52, and CL127 and with a monomer of 170 bp in length, was not associated with functional centromeres. In addition, clear pericentromeric heterochromatic domains were observed in several WL chromosomes. CL2-5bp is the second most abundant satDNA repeat found in most chromosomes that showed a mid-level association with functional centromeres. The third most abundant family of satDNA is comprised of CL10-78bp, CL21-38bp, and CL118-182bp. However, these three clusters share sequence similarities that clearly diverge. CL10-78bp and CL21-38bp share more similarities since the first is a dimer form of the latter, and both show association with functional centromeres. In contrast, CL118-182bp was not visualized by FISH, and its annotation on the genome revealed only small arrays more distally located.

We found that only two of the 10 most abundant satellite repeats occurred in the genome exclusively as long tandem arrays typical of satDNA. Both occupied specific genome regions. CL1-170bp was associated with pericentromeric knobs in a few chromosomes, extending to very large regions (e.g., LalbChr14 and LalbChr15) or even present as two distant in the pericentromeric regions of both chromosome arms but excluded from the centromeric regions (e.g., LalbChr13 and LalbChr24). Sometimes CL1-170bp arrays were truncated by other satDNAs or retrotransposons. The other case was CL53-24bp, which occurred also as long arrays closer to centromere-associated repeats, either centromeric satDNA or CRWL.

### Centromeric Satellite DNA Acts as Preferential Insertion Sites for Centromeric Retrotransposon of WL

As we have shown, a narrow and concentrated distribution of CRWL was observed in most assembled centromeres. A high association of CRWL and centromeric satellite DNA peaks was also observed, suggesting a more specific distribution of these repeats compared to other TEs. Furthermore, CRWL insertions were more frequently observed within CENH3-enriched cenDNA repeat arrays, which is also corroborated by FISH results, suggesting that cenDNA repeats may act as preferential insertion sites for CRWL. Common across several plants, CRMs tend to preferentially be inserted into functional centromeres (Neumann et al., 2011).

### Assembly Quality Assessment Based on Repeat Mapping

Remarkably, some chromosome assemblies lacked a large region of repeats, for instance, the Lalb_Chr01, which lacks a large pericentromeric region containing the CL1-170bp and another region that is most likely the functional centromere containing both CL2-5bp and CL10-78bp. The final assembly showed only the repeat CL53-24bp in the pericentromeric region of this chromosome, which is highly enriched in this chromosome. In fact, a gap in the assembly is observed upstream from the largest array of the CL53-24bp satDNA (10,697,827–10,788,322). Based on our repeat mapping results and taking into account that the 64 unplaced contigs from the WL assembly are mostly enriched with repeats, it is likely that some of these contigs represent fragmented peri- and centromeric regions.

Surprisingly, large arrays of CL2-5bp were rarely found on the reference genome of WL, contrasting to the bright signals observed for this repeat on most centromeric regions. This could be caused by a potential assembly failure and/or, less likely, by missing long-read sequencing coverage of this particular repeat. The reason the assembly/sequencing failed within arrays of this particular repeat is unknown, possibly the very short monomer size and high homogenization could have hampered its assembly/sequencing. For the CL55-8bp satDNA, which showed the highest enrichment in the CENH3-ChIPseq analysis, we observed signals in three chromosome pairs, but only two short arrays were found in the reference genome assembly, on Lalb_Chr07 and Lalb_Chr21.

## Conclusion

With the advancement of long-read sequence technologies, assembly of high-quality chromosome-scale reference genomes has become a feasible task for many non-model organisms. Here, we show that even the highly repeat-complex genome of WL has most of its repeats well assembled and validated based on our dense repeat FISH mapping strategy. Furthermore, the combination of a high-quality reference genome with a detailed FISH analysis of repeats allowed us to build a comprehensive chromosome-specific repeat profile of the WL genome. We also show the power of cytogenetics associated with genomics to solve gaps in the assembly and how it can support further improvement of the next versions of the reference genome. The highly repeat-diverse small genome of WL provides yet another good example of the rapid diversification of plant genomes. Considering the economic importance of several species of *Lupin* and the high number of species found in the genus, it will be of high interest to perform comparative cytogenomic analysis in the future. Furthermore, WL is a promising crop that could help increase plant protein as part of our diet and that will attract more and more interest from breeders in the near future. Focusing on the genomics of this ancient crop will certainly help meet this ambitious goal.

## Materials and Methods

### Plant Material for Cytological Analysis

Seeds of *L. albus* cv. AMIGA were germinated on Petri dishes with humid paper. After germination, the root tips were collected. For meiotic tissue, young flower buds were collected from flowering *L. albus* cv. AMIGA plants growing under controlled conditions in the greenhouse of the SupAgro—Montpellier. Pre-treatment and fixation of tissues were performed as described below.

### Chromosome Preparation for *in situ* Hybridization

Chromosome preparations for *in situ* hybridization analysis were conducted as described in Marques et al. (2015), with modifications. First, young roots (pre-treated with 8-hydroxyquinoline 2 mM for 3–5 h at room temperature) and anthers were fixed in 3:1 (ethanol:acetic acid) for 2–24 h. The fixed tissues were treated with an enzyme mixture (0.7% cellulase R10, 0.7% cellulase, 1.0% pectolyase, and 1.0% cytohelicase in 1 × citric buffer) for 1 h at 37°C. The material was then washed twice in water and fragmented in 7 μl of 60% freshly prepared acetic acid into smaller pieces with the help of a needle on a slide. Then another 7 μl of 60% acetic acid was added and the specimen was kept for 2 min at room temperature. Next, a homogenization step was performed with an additional 7 μl of 60% acetic acid and the slide was placed on a 55°C hot plate for 2 min. The material was spread by hovering a needle over the drop without touching the hot slide. After spreading the cells, the drop was surrounded by 200 μl of ice-cold, freshly prepared 3:1 (ethanol:acetic acid) fixative. More fixative was added and the slide was briefly washed in the fixative, then dipped in 60% acetic acid for 10 min, and dehydrated in 96% ethanol. The slides were stored until use in 96% ethanol at 4°C.

### Repeat Identification and Genome-Wide Repeat Annotation

Identification and characterization of moderately to highly repeated genomic sequences were achieved by graph-based clustering of genomic Illumina reads using RepeatExplorer2 pipeline (Novak et al., 2013). A total of 1,144,690 of 150-bp paired reads, representing ∼0.5 × genome coverage, were used for the clustering and 145 largest clusters with genome proportions of at least 0.01% were examined in detail. Clusters containing satellite repeats were identified based on the presence of tandem sub-repeats within their read or assembled contig sequences with TAREAN (Novak et al., 2017). Genome-wide repeat annotation was performed using the DANTE (Domain-based ANnotation of Transposable Elements) tool (Neumann et al., 2019), and the GFF3 file generated was manually edited on Geneious software v.9.1.3 and further incorporated on the full *L. albus* genome annotation (see text footnote 1).

### Probe Preparation and Fluorescence *in situ* Hybridization

Fluorescence *in situ* hybridization (FISH) probes were obtained as 5′–Cy3 or 5′–FAM-labeled oligonucleotides (Eurofins MWG Operon),^2^ or were PCR-amplified as described below. All DNA probes, except oligonucleotides, were labeled with Cy3- or Alexa 488-dUTP (Jena Bioscience) by nick translation, as described by Kato et al. (2006). The sequences of all oligonucleotides and primers are listed in Supplementary Table 2. FISH was performed as described in Marques et al. (2015). Probes were then mixed with the hybridization mixture (50% formamide and 20% dextran sulfate in 2 × SSC), dropped onto slides, covered with a coverslip, and sealed. After denaturation on a heating plate at 80°C for 3 min, the slides were hybridized at 37°C overnight. Post-hybridization washing was performed in 2 × SSC for 20 min at 58°C. After dehydration in an ethanol series, 4′,6–diamidino-2–phenylindole (DAPI) in Vectashield (Vector Laboratories)^3^ was applied. Microscopic images were recorded using a Zeiss Axiovert 200M microscope equipped with a Zeiss AxioCam CCD. Images were analyzed using the ZEN software (Carl Zeiss GmbH).

### PCR Amplification of Tandem Repeat and Retroelement Fragments for Probe Labeling

Fragments for probe labeling were amplified using gDNA from *L. albus* using the forward and reverse primers, as given in Supplementary Table 2. Eight PCR reactions for each target repeat were performed in 50 μl reaction volume containing 100 ng of gDNA, 1 μM primers, 1 × PCR buffer, 0.2 mM dNTPs, and 1U of Taq polymerase (Qiagen). Thirty-five amplification cycles with proper conditions for each set of primers were run. PCR reactions were sampled, purified, and concentrated using Wizard^®^ SV Gel and PCR Clean-Up System (Promega). Sanger sequencing confirmed the correct amplification of PCR fragments. After confirmation, the PCR products containing the same class of repeat were collected and used for probe labeling by nick translation as described above.

### Chromatin-Immunoprecipitation Sequencing (ChIPseq) Analysis

CENH3-ChIPseq was previously generated, as in Hufnagel et al. (2020), and used in this study to revisit the enrichment profile of CENH3 along each repeat family localized in the pericentromeric chromatin of WL. Repeat family-specific enrichment for CENH3 association was performed with deeptools2 implemented in the Galaxy platform (Ramirez et al., 2014, 2016).

ChIPseq Illumina paired-end reads were mapped to WL genome assembly (cv. AMIGA) (Hufnagel et al., 2020) using Bowtie2 version 2.4.4 (Langmead and Salzberg, 2012) with default parameters. For subsequent analysis only reads with *q* > 30 were used. Read coverage BIGWIG files were generated from original BAM files using the bamCoverage tool of the deepTools suite (Ramirez et al., 2016) with the options: centerReads. Heatmap plotting was done by computeMatrix and plotHeatmap tools available in deeptools2. For plotting at repeat regions in the genome, we used RSS and repeat end sites (RES). The RSS used are all repeat start points for each repeat family separated from repeat annotated genome assembly generated as mentioned above. Similarly, the RES used are all repeat end points.

### Centromere *in silico* Characterization

Based on our previous CENH3-ChIP analysis (Hufnagel et al., 2020), the regions surrounding and containing the CENH3-associated DNA repeats were isolated for a fine-scale characterization of their repeat composition and organization. Detection of the retrotransposon protein-coding domains in the individual centromeric regions was performed using DANTE, which is a bioinformatic tool available on the RepeatExplorer server^4^ employing the LAST program (Kielbasa et al., 2011) for similarity searches against the REXdb protein database (Neumann et al., 2019). Satellite repeat sequences and rDNA loci were annotated in individual pericentromeric regions of the reference WL genome using the Geneious annotation tool by similarity searches against a reference database compiled from contigs assembled from clusters of WL Illumina reads in the frame of our previous study (Hufnagel et al., 2020). Additionally, the database included consensus sequences, and their most abundant sequence variants were calculated from the same Illumina reads using the TAREAN pipeline (Novak et al., 2017). Finally, annotated centromeric regions were subjected to dot–plot analysis with FlexiDot software (Seibt et al., 2018) for further characterization of high-order structure.

### Graphical Visualization of Annotation and Ideograms

The graphical distribution of annotated TEs on the reference chromosomes of WL was done using the IGB program (Nicol et al., 2009) with the generated DANTE GFF annotation files. The graphical distribution of annotated satellite repeats on the reference chromosomes of WL was done using DensityMap Perl script^5^ with satellite GFF3 annotation files.

## Data Availability Statement

The datasets presented in this study can be found in online repositories. The names of the repository/repositories and accession number(s) can be found in the article/Supplementary Material.

## Author Contributions

AM, BH, and AS performed all experiments. AM and BP designed the research. AM, BH, and BP wrote the manuscript. All authors contributed to the article and approved the submitted version.

## Conflict of Interest

The authors declare that the research was conducted in the absence of any commercial or financial relationships that could be construed as a potential conflict of interest.

## Publisher’s Note

All claims expressed in this article are solely those of the authors and do not necessarily represent those of their affiliated organizations, or those of the publisher, the editors and the reviewers. Any product that may be evaluated in this article, or claim that may be made by its manufacturer, is not guaranteed or endorsed by the publisher.

## Footnotes

1
www.whitelupin.fr

2
http://www.eurofinsdna.com

3
http://www.vectorlabs.com

4
https://repeatexplorer-elixir.cerit-sc.cz/

5
https://github.com/sguizard/DensityMaps
