# Supplementary material for: The Highly Repeat-Diverse (Peri) Centromeres of White Lupin (Lupinus albus L.)
Source: Front Plant Sci. 2022 Apr 5;13:862079. doi: 10.3389/fpls.2022.862079 (PMC9016224; doi:10.3389/fpls.2022.862079)
Supplement: Supplementary file 1 [file Data_Sheet_1.PDF]

Supplementary Tables and Figures

Suppl. Table 1. Repeat composition of white lupin genome (Hufnagel et al. 2020).

<table>
<tr><th colspan="3">Class</th><th>Genomic abundance [%]</th></tr>
<tr><td colspan="2" rowspan="6">LTR retroelements Ty1/copia</td><td>Ale</td><td>0.18</td></tr>
<tr><td>Ikeros</td><td>1.21</td></tr>
<tr><td>Ivana</td><td>0.20</td></tr>
<tr><td>SIRE</td><td>6.26</td></tr>
<tr><td>TAR</td><td>0.43</td></tr>
<tr><td>Tork</td><td>0.96</td></tr>
<tr><td rowspan="4">LTR retroelements Ty3/gypsy</td><td rowspan="2">non-chromovirus</td><td>Athila</td><td>1.24</td></tr>
<tr><td>Ogre</td><td>0.27</td></tr>
<tr><td rowspan="2">chromovirus</td><td>CRM</td><td>3.40</td></tr>
<tr><td>Tekay</td><td>16.68</td></tr>
<tr><td colspan="2" rowspan="4">DNA transposons</td><td>EnSpm CACTA</td><td>0.34</td></tr>
<tr><td>hAT</td><td>0.04</td></tr>
<tr><td>MuDR Mutator</td><td>0.27</td></tr>
<tr><td>Helitron</td><td>0.11</td></tr>
<tr><td colspan="3">Other TEs</td><td>11.64</td></tr>
<tr><td colspan="2" rowspan="2">rDNA</td><td>35S rDNA</td><td>2.43</td></tr>
<tr><td>5S rDNA</td><td>0.15</td></tr>
<tr><td colspan="3">Tandem repeats</td><td>14.71</td></tr>
<tr><td colspan="3">Total</td><td>60.52</td></tr>
</table>

Suppl. Table 2. Primers and Oligo sequences used.

Sequence type	Primer name	Sequence	Obs.:
satDNA	CL1-170bp-F1	GTGACATATGCATTTGGGGTAG	
satDNA	CL1-170bp-R1	ACGTCCAATTCTGATATACGGAA	
satDNA	CL10-78bp-F	AAAASTAGCCACAAARAAGT	centromeric DNA
satDNA	CL10-78bp-R	MAGCACAAATAACTCCCAA	centromeric DNA
satDNA	CL77-36bp-F	TTTTATCGTCTCGAGCCCGAC	
satDNA	CL77-36bp-R	ATAAATATGCGAGCATGTCGGG	
satDNA	CL85-76bp-F	GCCTTAGCAAATGAAAATGGTTGT	
satDNA	CL85-76bp-R	GCTAAGGCATTAGTATACCATTTTCT	
satDNA	CL114-247bp-F	CCYAAAACCATTTTYWGGAAC	degenerated primer
satDNA	CL114-247bp-R	ATGGTTTTRGGTTTGYCTTGA	degenerated primer
satDNA	CL118-182bp-F	ATAAGCCCACAATTTATCTGTGGT	
satDNA	CL118-182bp-R	GCACATAAAAAGCTCTCCCTCG	
satDNA	CL121-918bp-F	GTGCTATTGTGAAGTACGTGTTT	
satDNA	CL121-918bp-R	CCTCAACATTTTCAAAAGCTAACG	
LTR	INT-Tekay-SC1-F1	CAGAAAGCCAAGATTGAGCATC	
LTR	INT-Tekay-SC1-R1	CCCAATATCAAGTTCTCCCCAG	
LTR	RT-Tekay-SC1-F1	GTGCGCCTGTTTTGTTGGTT	
LTR	RT-Tekay-SC1-R1	TGTTCCTCTCGGCTTTTGGAG	
LTR	INT-CRM-SC5-F1	CATGGGCTTTACACGCCCTT	centromeric DNA
LTR	INT-CRM-SC5-R1	GTAAGTCCAGCGGGGTCAAA	centromeric DNA
LTR	RT-CRM-SC5-F1	CAGAGCATGAGCCCTTGTG	centromeric DNA
LTR	RT-CRM-SC5-R1	TGATCCACGCAAAAGGAACA	centromeric DNA
LTR	RT-CRM-SC5-F2	CAACAGAGCATGAGCCCTTG	centromeric DNA
LTR	RT-CRM-SC5-R2	TGACATGATCCACGCAAAAGG	centromeric DNA
LTR	RT-SIRE-SC3-F1	ATTGAGGATGTGCACTGGGG	
LTR	RT-SIRE-SC3-R1	CCAAGCCTCTTTCGAACCCT	

LTR	INT-SIRE-SC3-F1	CGCGTTGCACACATCCATAT
LTR	INT-SIRE-SC3-R1	TTTGTTGTGGAGTGCGAGGA
LTR	INT-Ikeros-CL23-F1	AGCGTCTTGTGTCAGATGGG
LTR	INT-Ikeros-CL23-R1	CCTGGACGTTGTTCACCTGA
LTR	RT-Ikeros-CL23-F1	ACCAAGCGGGATTCTAACGG
LTR	RT-Ikeros-CL23-R1	TGTGCTTGCTCCCACTGAAT

Sequence type	Oligo-probe name	Sequence and labeling	fluorescent dye
satDNA	CL1-sat170bp	[Cy3]GTATATCMGAATTGGACGTRTGACATATGM	Cy3
satDNA	CL2-sat5bp	[Cy3]GGATAGGATAGGATAGGATAGGATA	Cy3
satDNA	CL10-sat78bp	[Cy3]MAAATWACTCCCAAACTTSTTTGTGGC	Cy3
satDNA	CL21-sat38bp	[FAM]GAAAAGTAGCCAAACAAACAAAAG	FAM
satDNA	CL53-sat24bp	[FAM]TGACTTACTTAATGAATGACTGAC	FAM
satDNA	CL77-sat36bp	[Cy3]GCTCDAGAYGATAAAATAAATATGYGA	Cy3
satDNA	CL55-sat8bp	[Cy3]CTAAACTCCTAAACTCCTAAACTCCT	Cy4
Telomere	CL68-Telomere-motif	[FAM]CTAAACCCTAAACCCTAAACCCTAAA	FAM

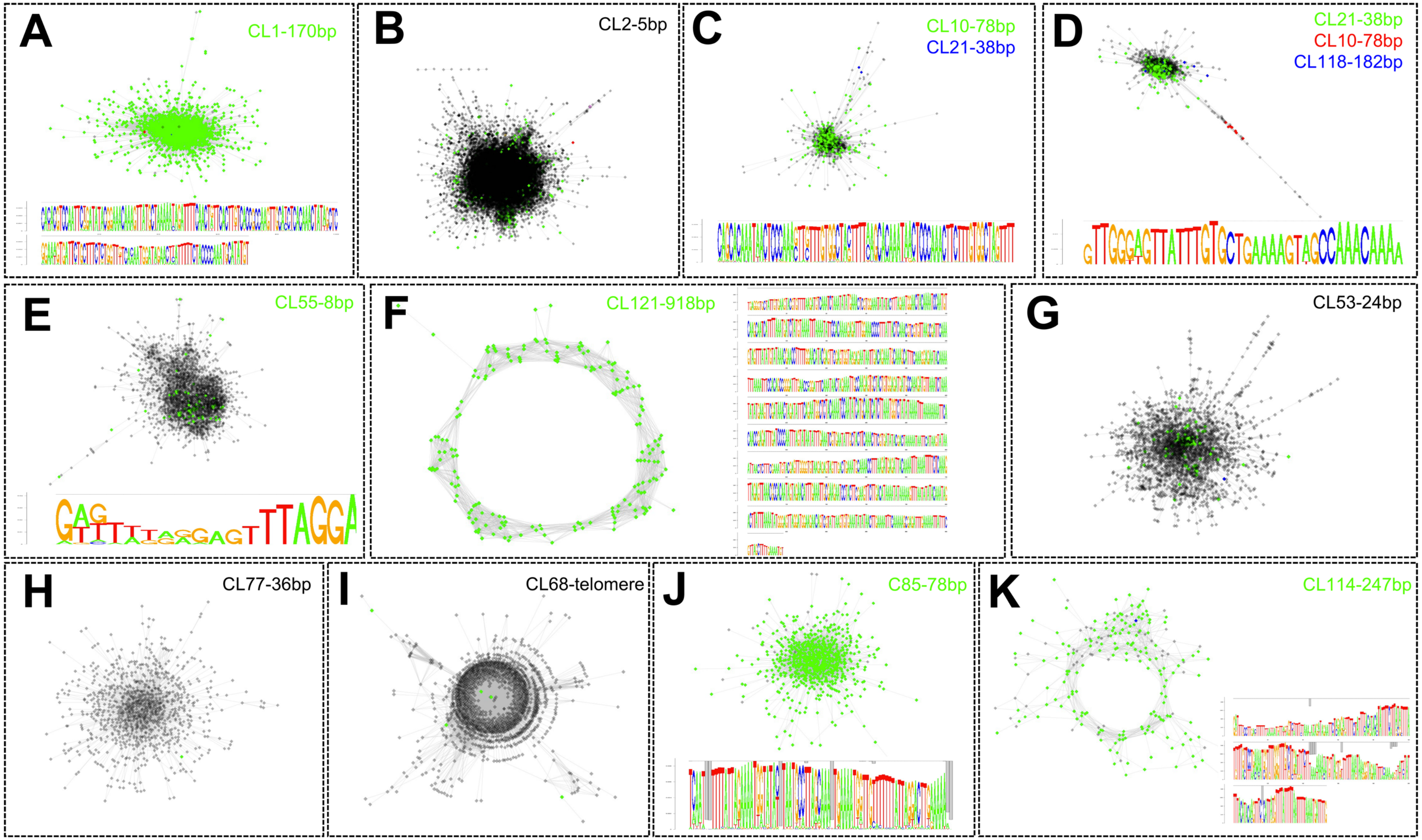

Suppl. Figure 1. RepeatExplorer2 clusters that were idenfied as tandem repeats (**A-K**). Sequence logos are presented only for the clusters that were initially identified as tandem repeats by the TAREAN tool from RepeatExplorer2.

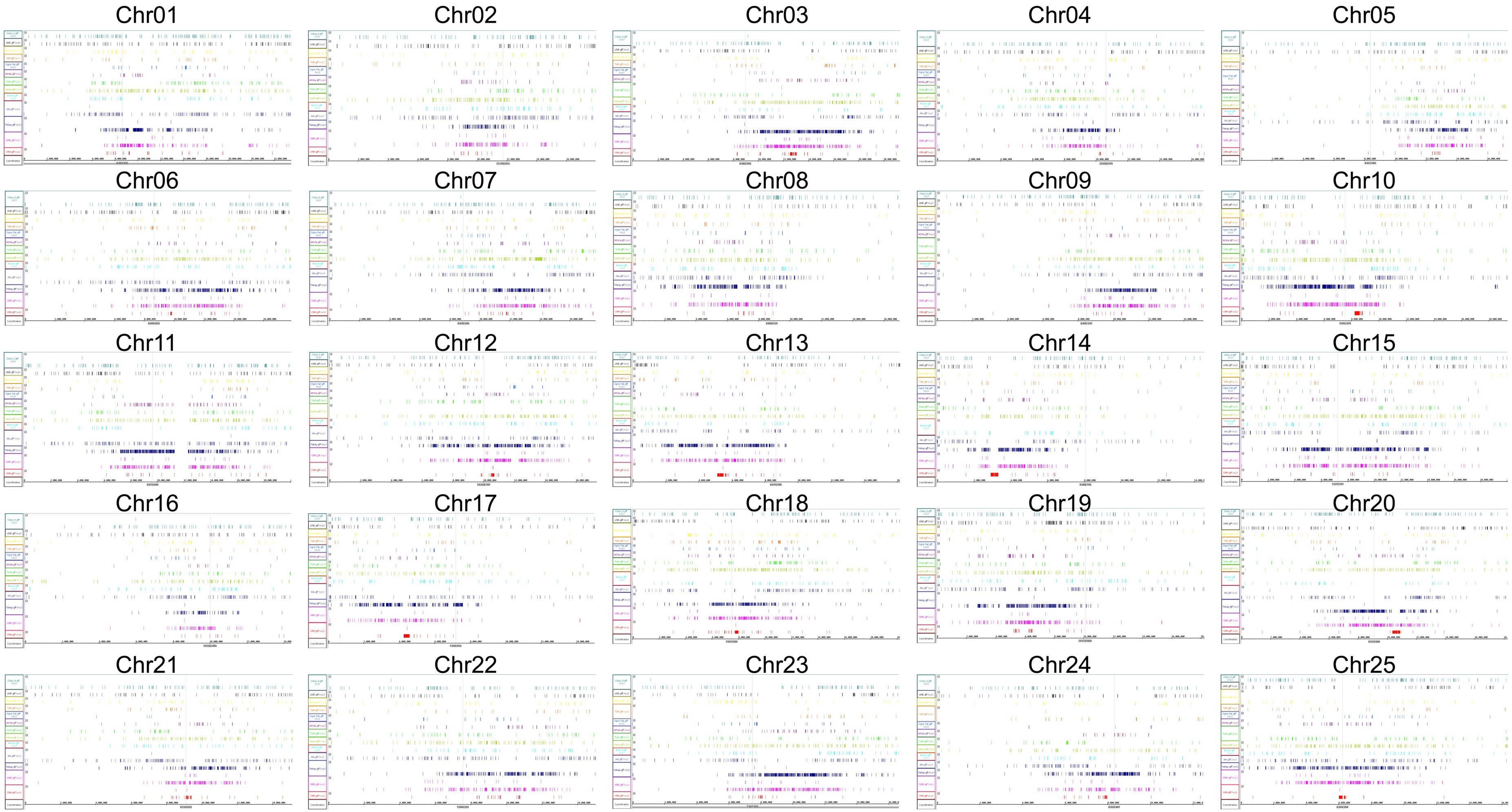

Suppl. Figure 2. Density profile all the main families of transposable elements of class I and II along each white lupin chromosome.

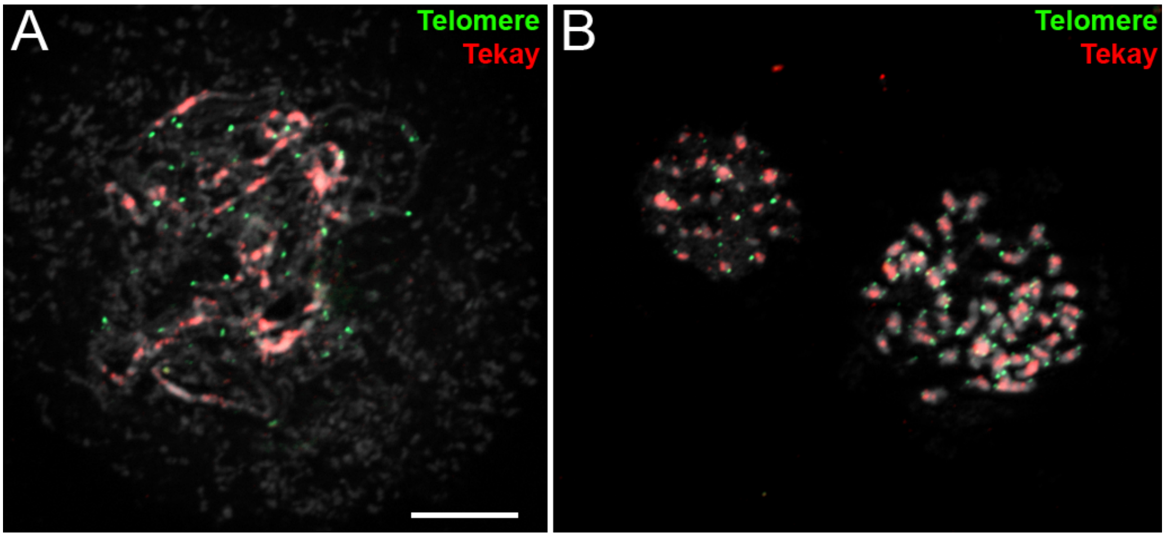

Suppl. Figure 3. FISH with Tekay and Telomeric (TTTAGGG)n probes showing an enrichment for Tekay along the pericentromeric heterochromatin of all chromosomes. (**A**) Pachytene. (**B**) Interphase nucleus (left) and mitotic metaphase (right). Scale bar = 10 µm.

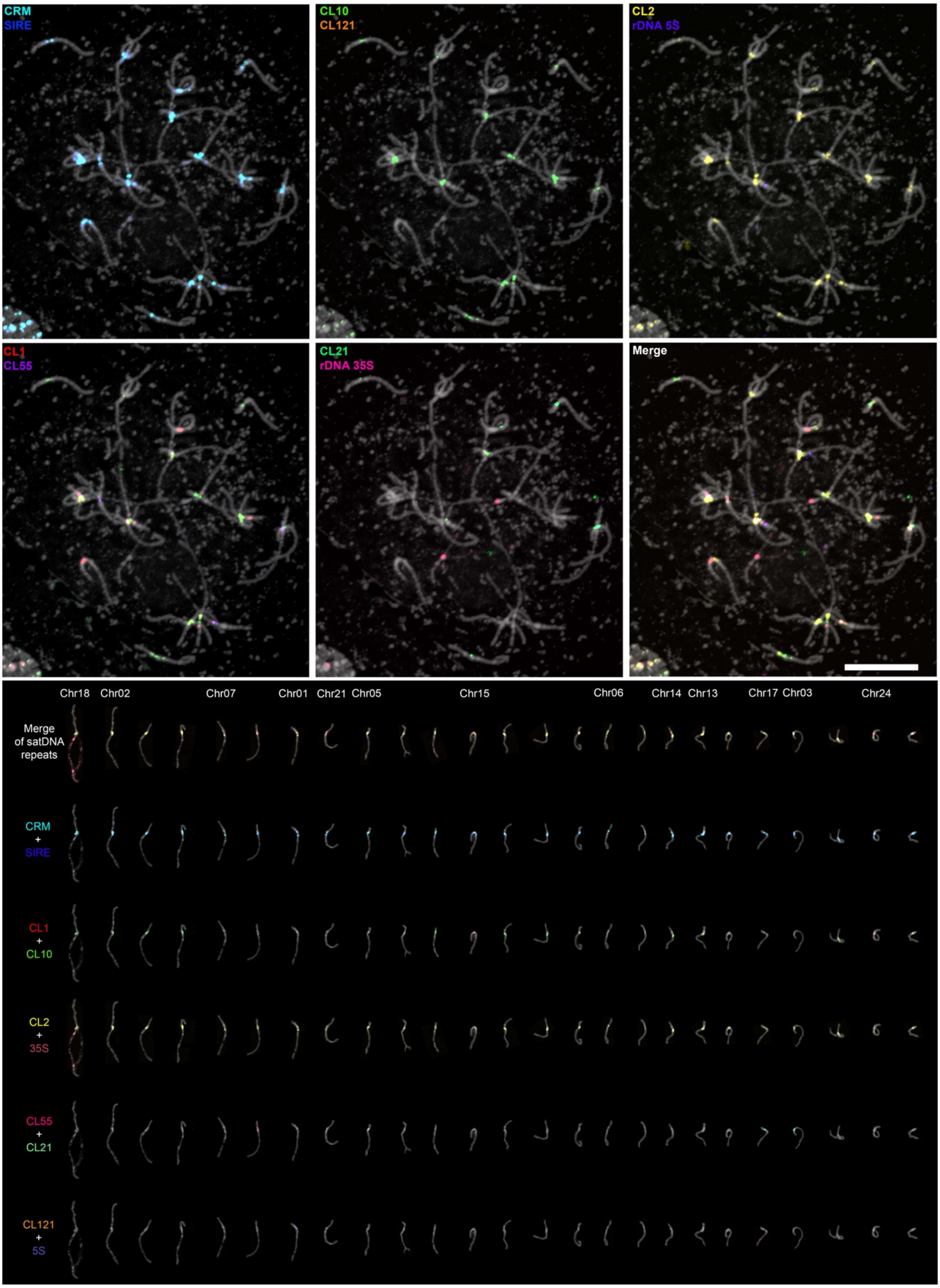

Suppl. Figure 4. Sequential Multicolor FISH in a pachytene cell of white lupin. Potentially assigned chromosomes are indicated. Scale bar = 10 µm.

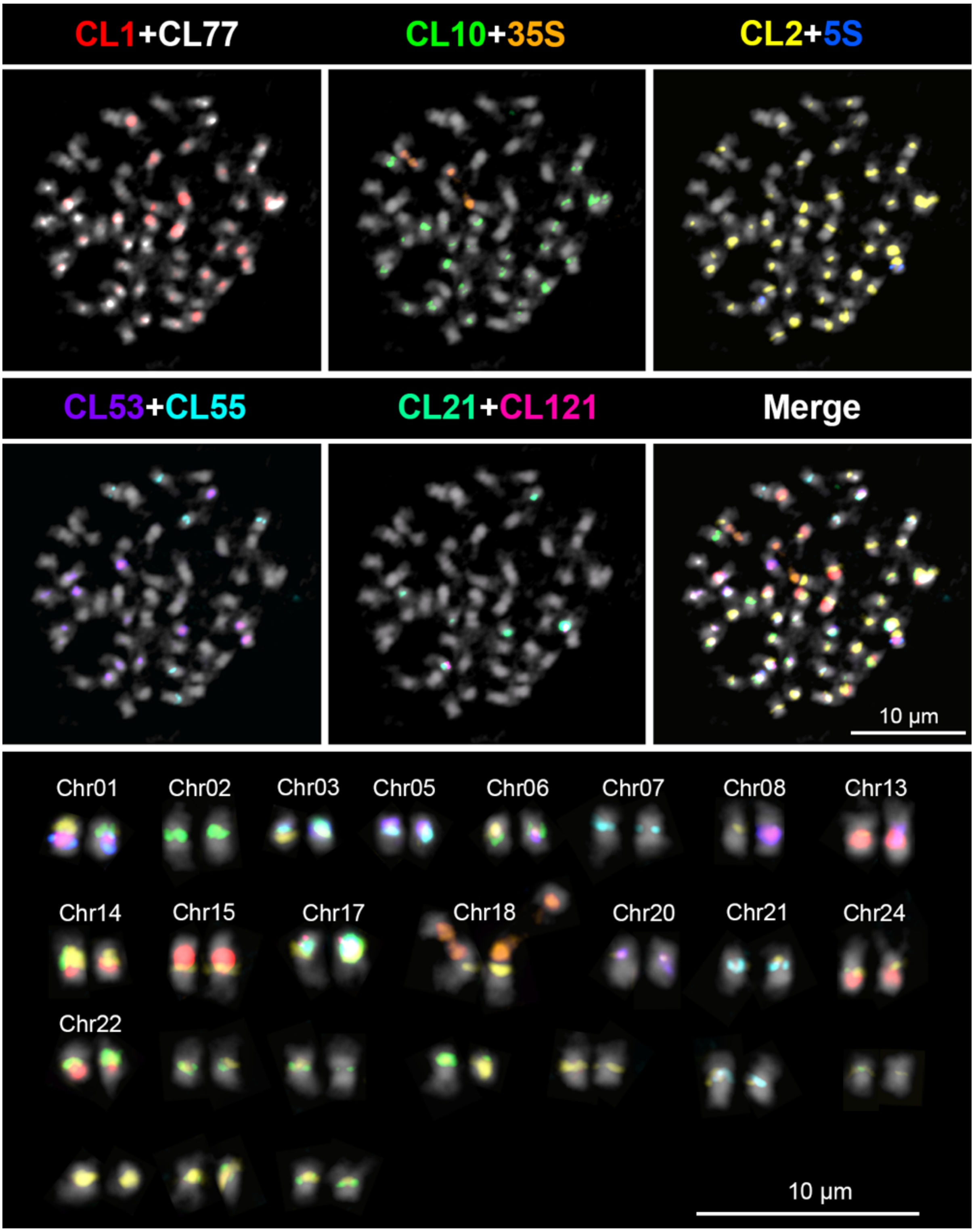

Suppl. Figure 5. Sequential Multicolor FISH in an additional mitotic metaphase cell of white lupin. Potentially assigned chromosomes are indicated. Scale bar = µm.
