# Supplementary material for: The Highly Repeat-Diverse (Peri) Centromeres of White Lupin (Lupinus albus L.)
Source: Front Plant Sci. 2022 Apr 5;13:862079. doi: 10.3389/fpls.2022.862079 (PMC9016224; doi:10.3389/fpls.2022.862079)
Supplement: Supplementary file 2 [file Presentation_1.pdf]

Supplementary Dataset 1

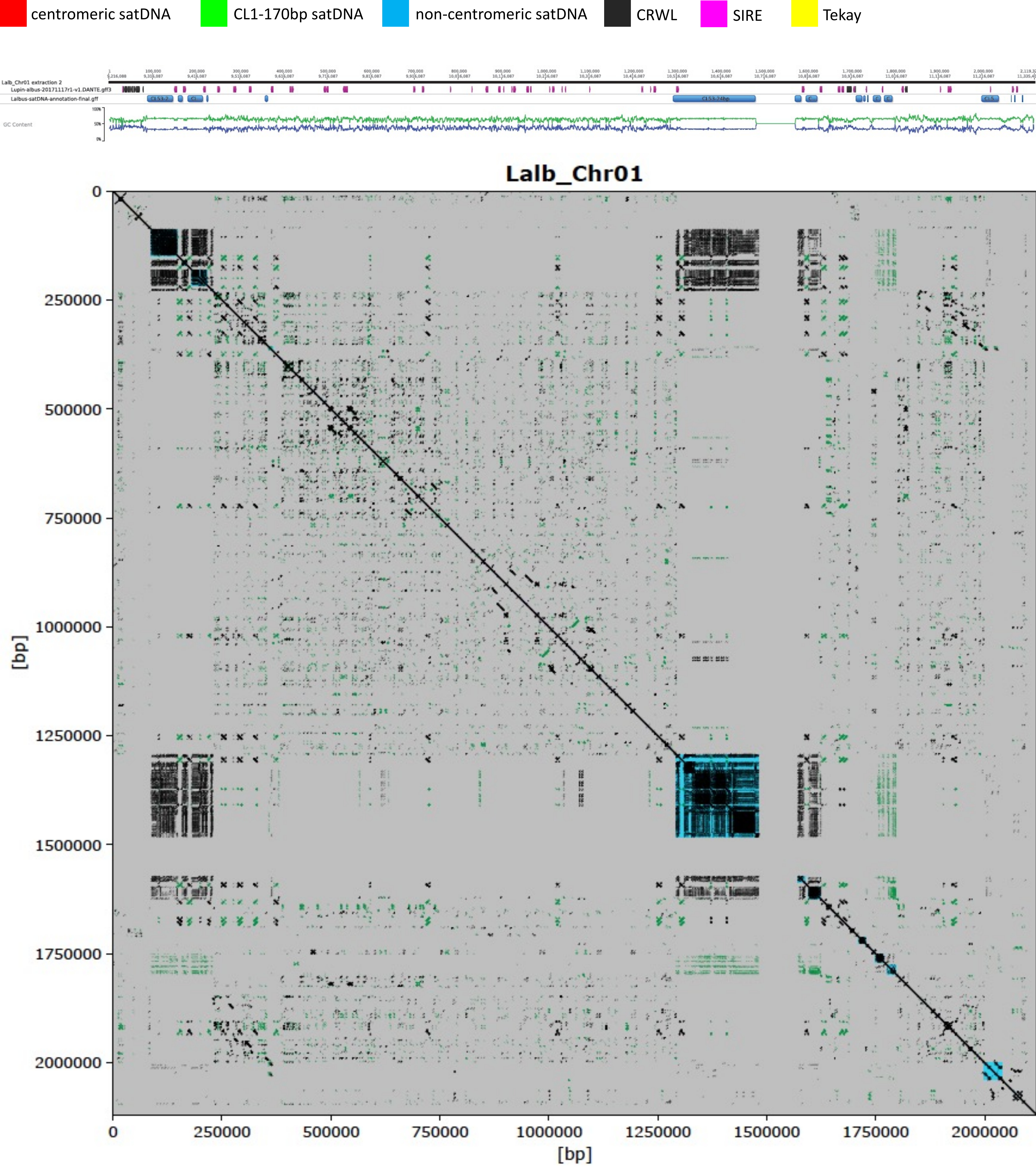

centromeric satDNA
CL1-170bp satDNA
non-centromeric satDNA
CRWL
SIRE
Tekay
Lalb_Chr01
[bp]
[bp]
0
250000
500000
750000
1000000
1250000
1500000
1750000
2000000

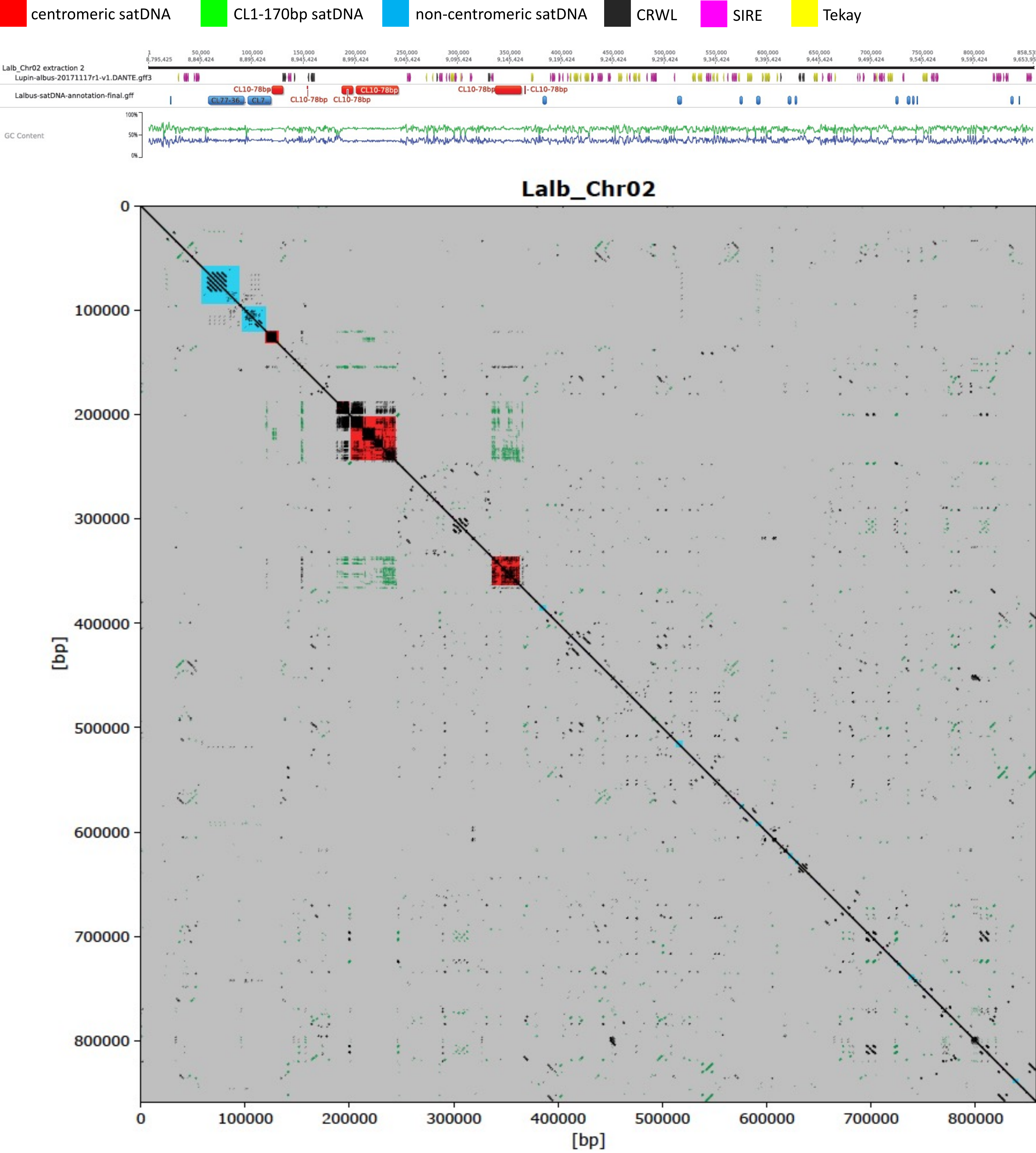

centromeric satDNA
CL1-170bp satDNA
non-centromeric satDNA
CRWL
SIRE
Tekay
Lalb_Chr02 extraction 2
Lupin-albus-20171117r1-v1.DANTE.gff3
Lalbus-satDNA-annotation-final.gff
GC Content
CL10-78bp
Lalb_Chr02
[bp]
[bp]

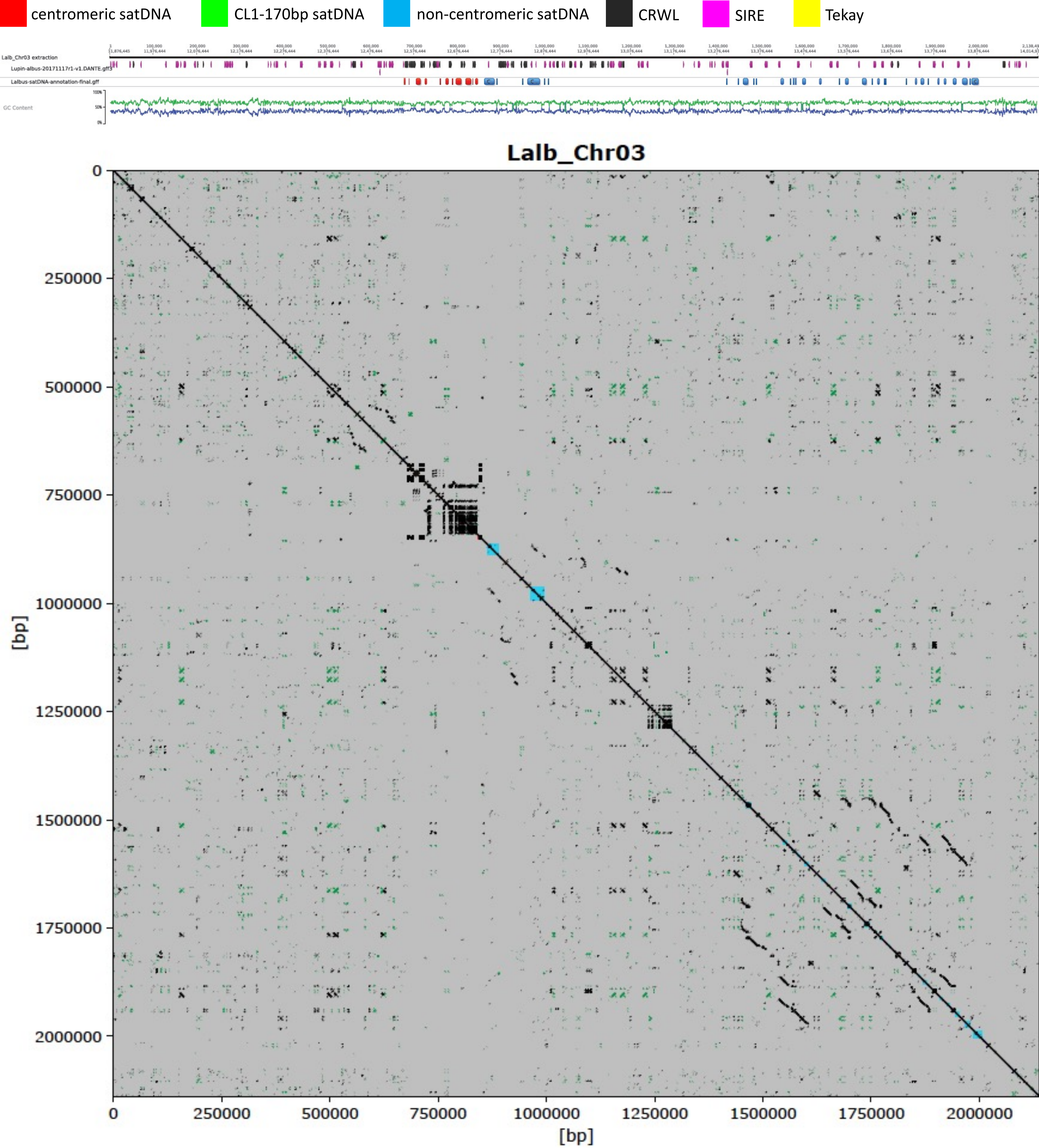

centromeric satDNA
CL1-170bp satDNA
non-centromeric satDNA
CRWL
SIRE
Tekay
Lalb_Chr03
0
250000
500000
750000
1000000
1250000
1500000
1750000
2000000
[bp]

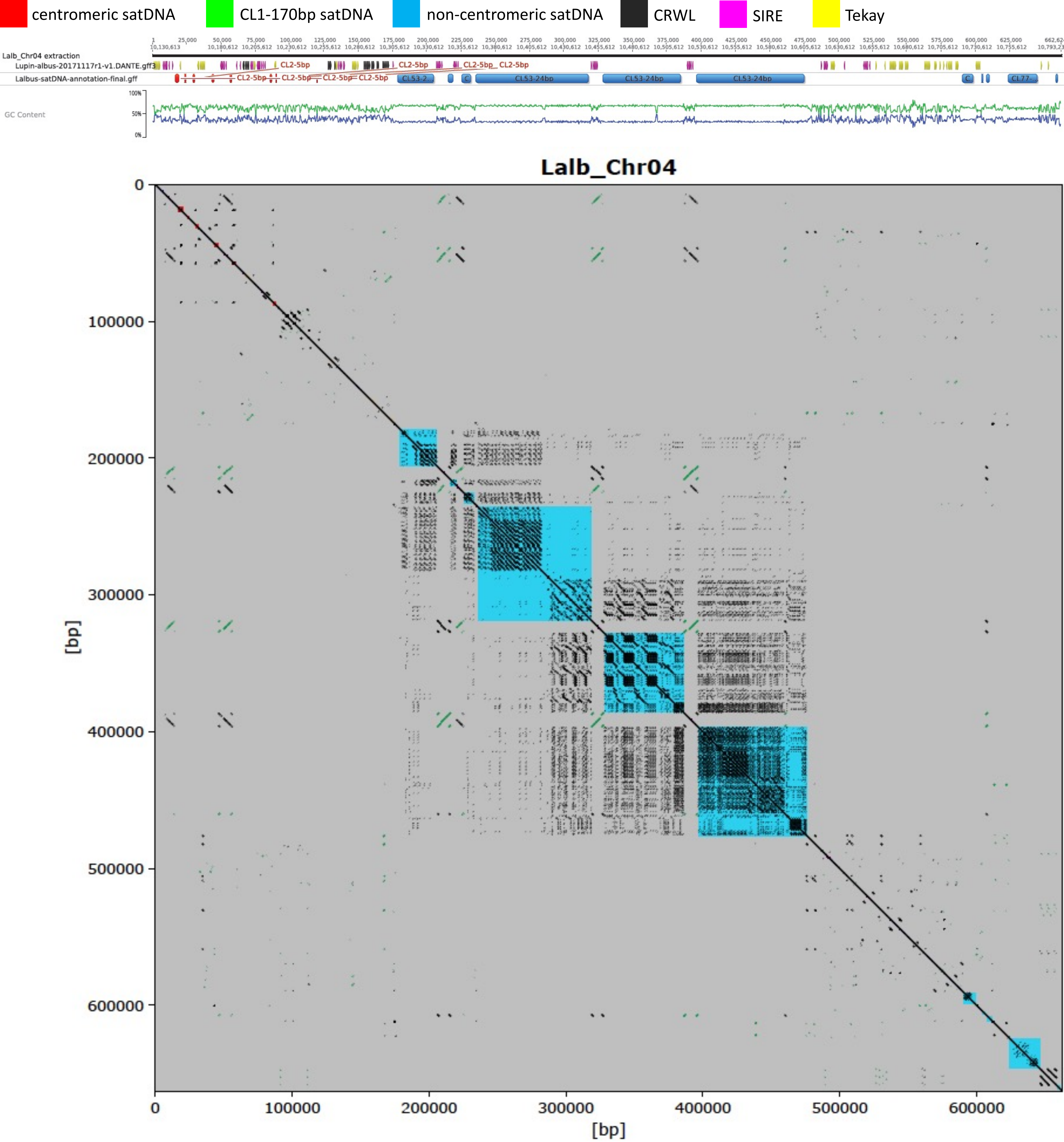

centromeric satDNA
CL1-170bp satDNA
non-centromeric satDNA
CRWL
SIRE
Tekay
Lalb_Chr04 extraction
Lupin-albus-20171117r1-v1.DANTE.gff3
Lalbus-satDNA-annotation-final.gff
GC Content
Lalb_Chr04
[bp]
[bp]
0
100000
200000
300000
400000
500000
600000

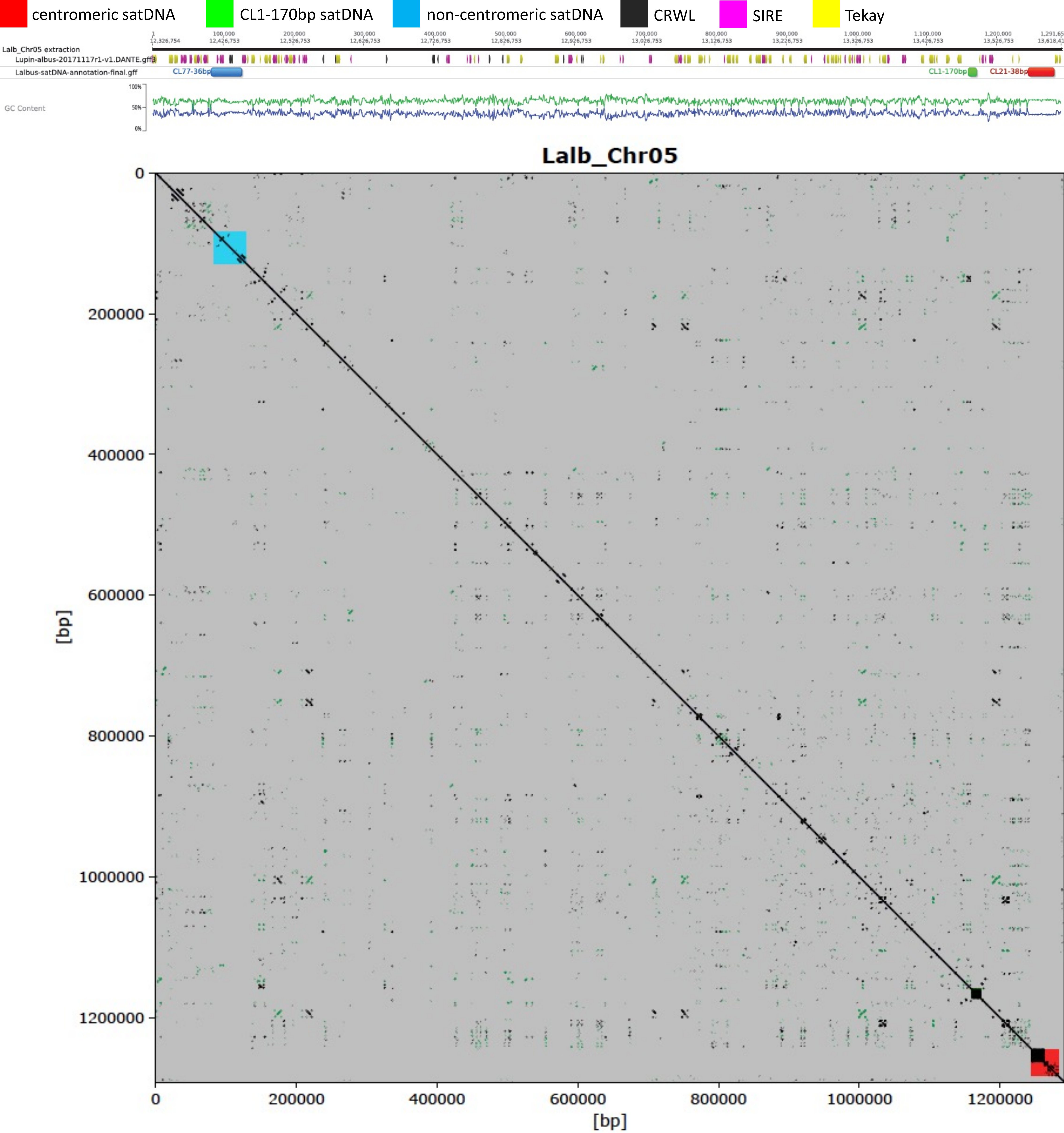

centromeric satDNA
CL1-170bp satDNA
non-centromeric satDNA
CRWL
SIRE
Tekay
Lalb_Chr05 extraction
Lupin-albus-20171117r1-v1.DANTE.gff3
Lalbus-satDNA-annotation-final.gff
CL77-36bp
CL1-170bp
CL21-38bp
GC Content
100%
50%
0%
Lalb_Chr05
0
200000
400000
600000
800000
1000000
1200000
[bp]
[bp]

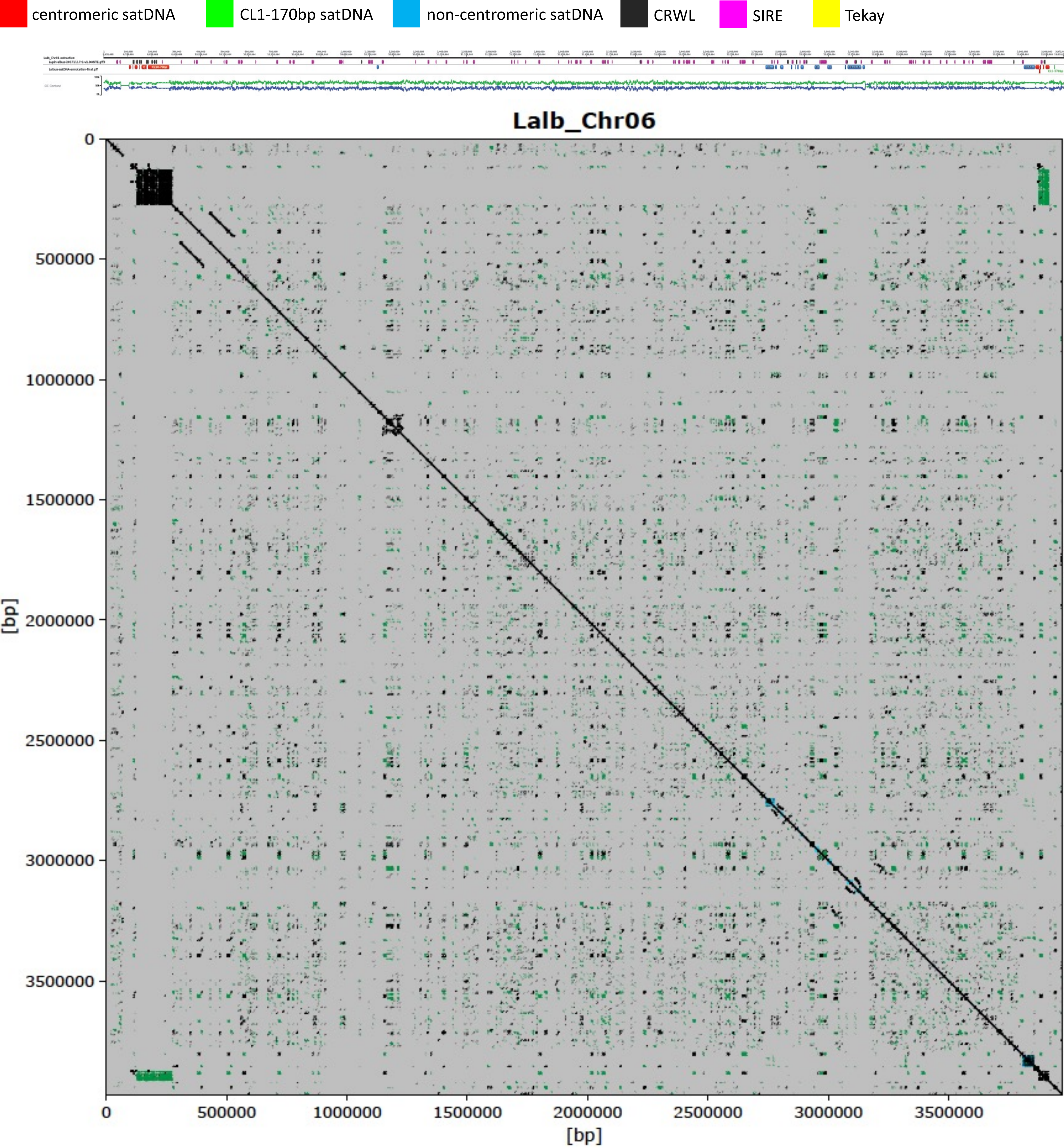
centromeric satDNA
CL1-170bp satDNA
non-centromeric satDNA
CRWL
SIRE
Tekay
Lalb_Chr06
0
500000
1000000
1500000
2000000
2500000
3000000
3500000
[bp]
0
500000
1000000
1500000
2000000
2500000
3000000
3500000
[bp]

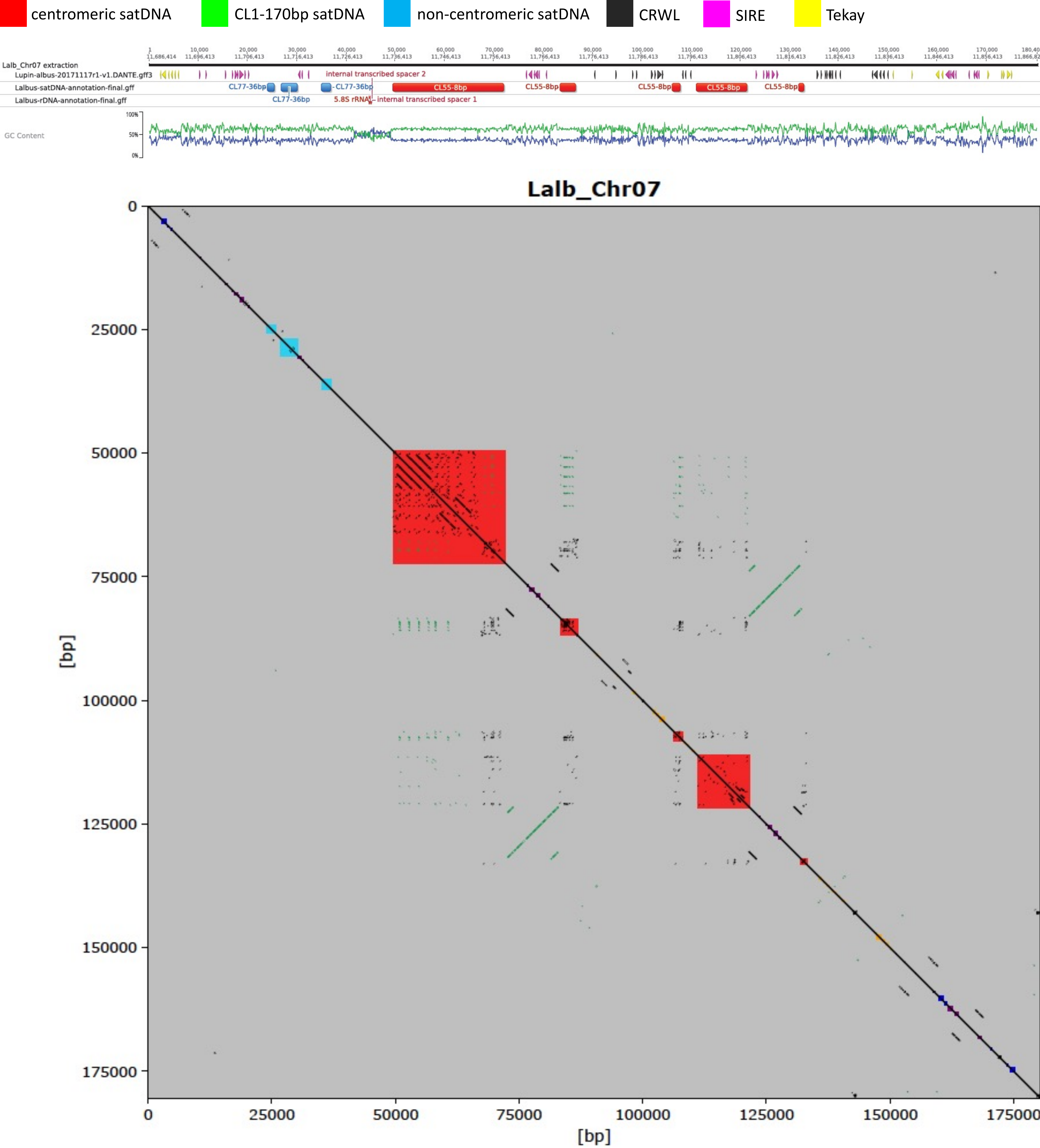

centromeric satDNA
CL1-170bp satDNA
non-centromeric satDNA
CRWL
SIRE
Tekay
Lalb_Chr07 extraction
Lupin-albus-20171117r1-v1.DANTE.gff3
Lalbus-satDNA-annotation-final.gff
Lalbus-rDNA-annotation-final.gff
internal transcribed spacer 2
CL77-36bp
CL55-8bp
5.8S rRNA
internal transcribed spacer 1
GC Content
Lalb_Chr07
[bp]
[bp]

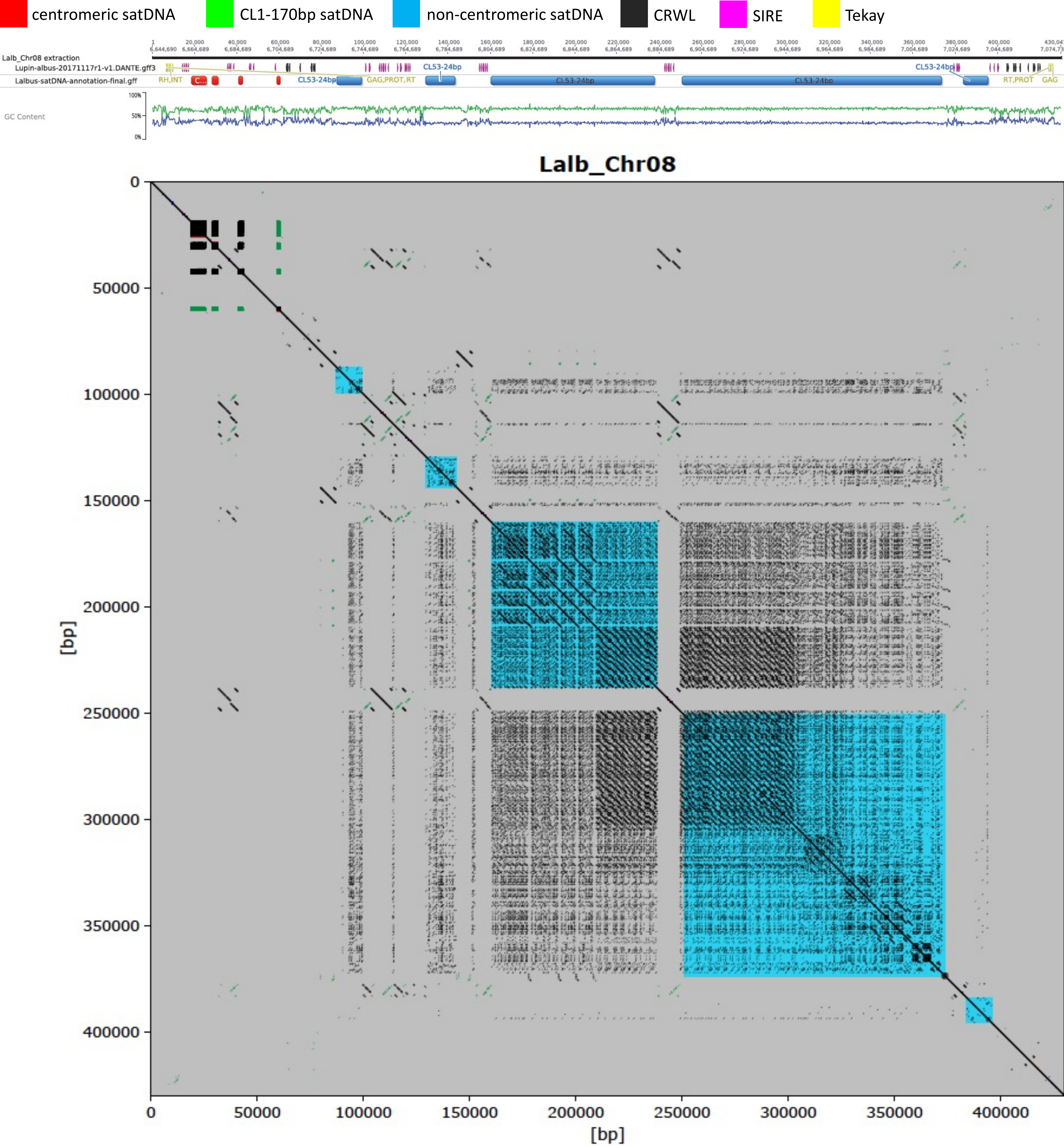

centromeric satDNA
CL1-170bp satDNA
non-centromeric satDNA
CRWL
SIRE
Tekay
Lalb_Chr08 extraction
Lupin-albus-20171117r1-v1.DANTE.gff3
Lalbus-satDNA-annotation-final.gff
GC Content
Lalb_Chr08
[bp]
[bp]

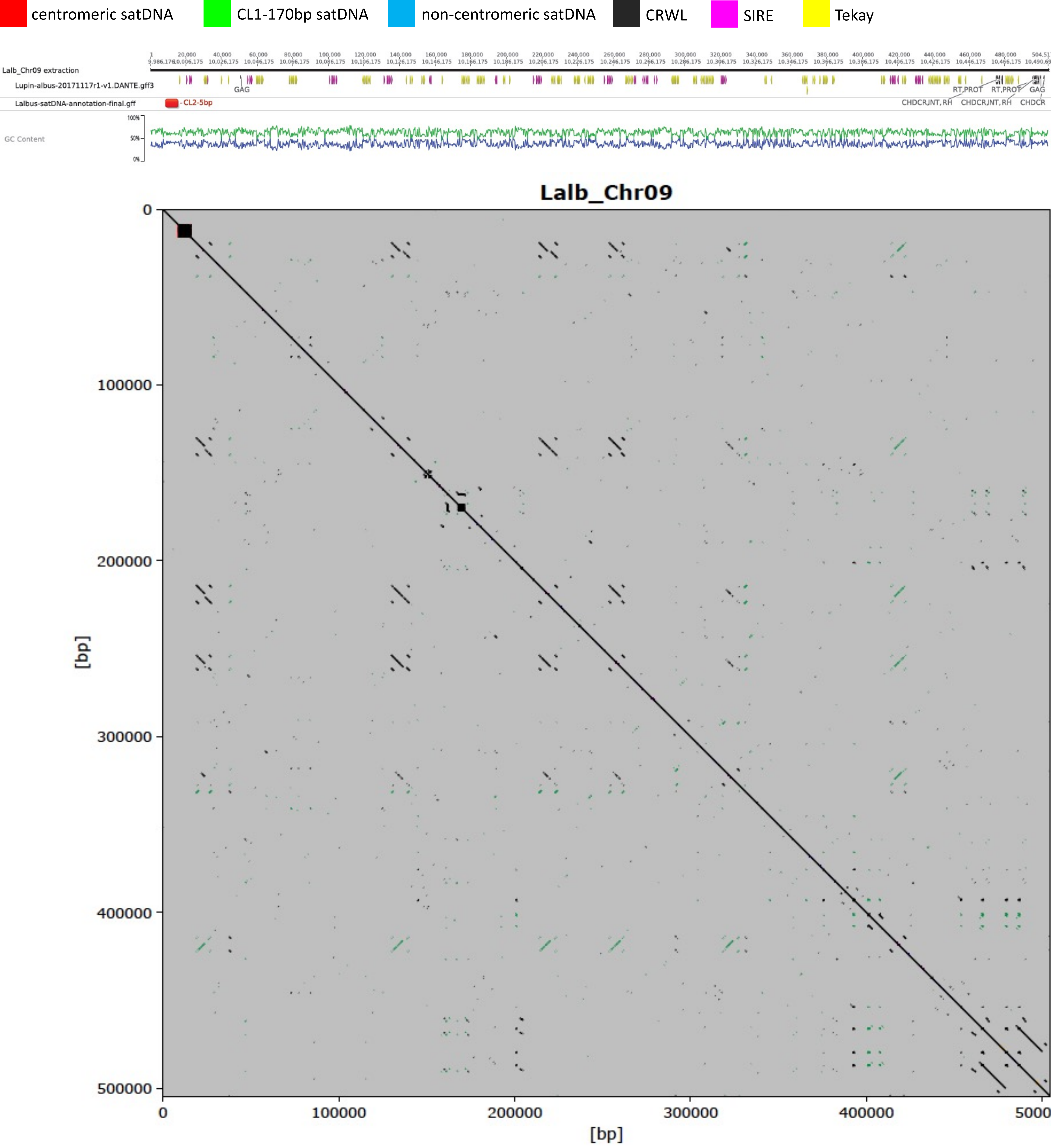
centromeric satDNA
CL1-170bp satDNA
non-centromeric satDNA
CRWL
SIRE
Tekay
Lalb_Chr09 extraction
Lupin-albus-20171117r1-v1.DANTE.gff3
Lalbus-satDNA-annotation-final.gff
CL2-5bp
GC Content
Lalb_Chr09
[bp]
[bp]

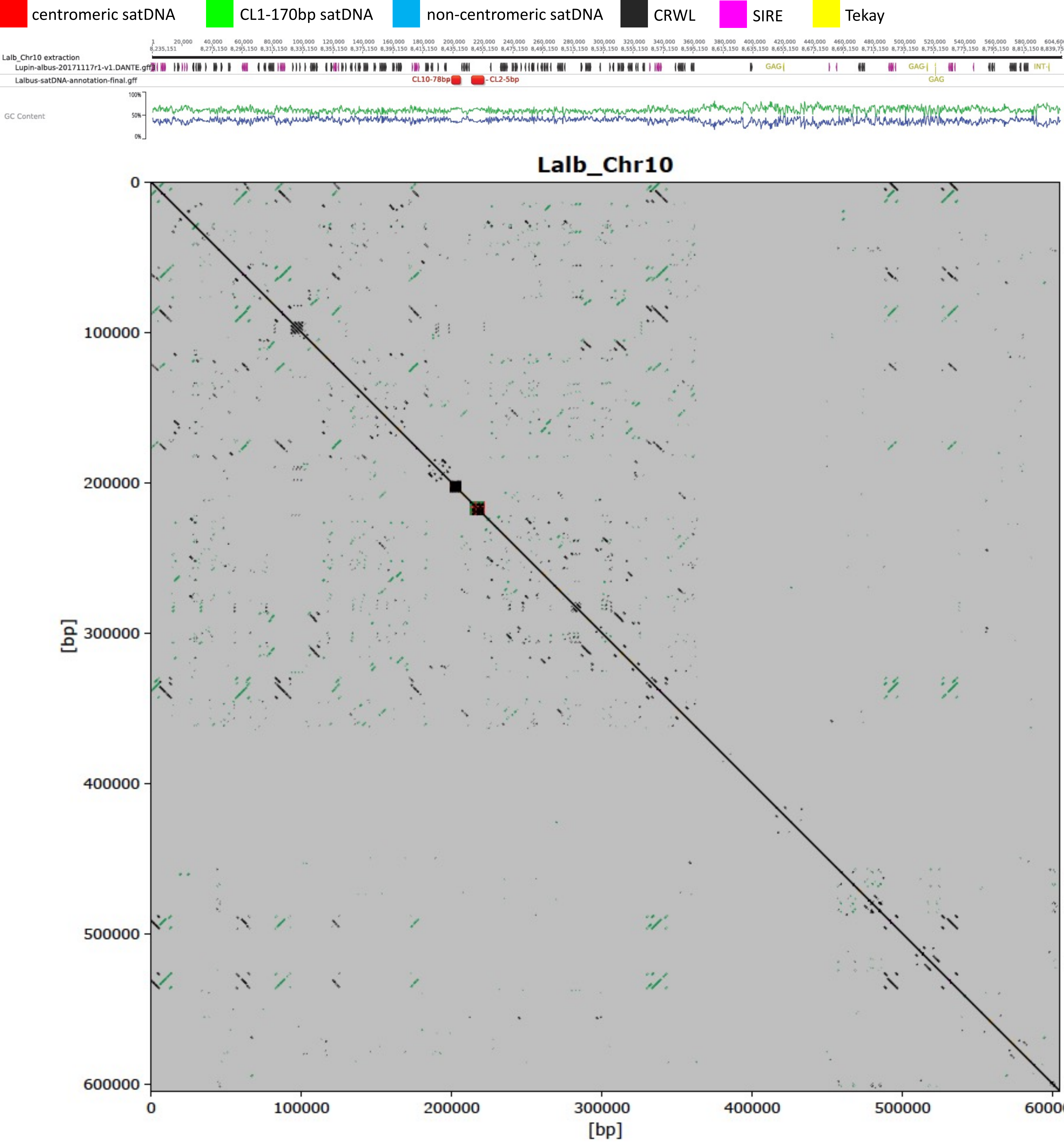
centromeric satDNA
CL1-170bp satDNA
non-centromeric satDNA
CRWL
SIRE
Tekay
Lalb_Chr10 extraction
Lupin-albus-20171117r1-v1.DANTE.gff3
Lalbus-satDNA-annotation-final.gff
CL10-78bp
CL2-5bp
GAG
INT
GC Content
Lalb_Chr10
[bp]
[bp]

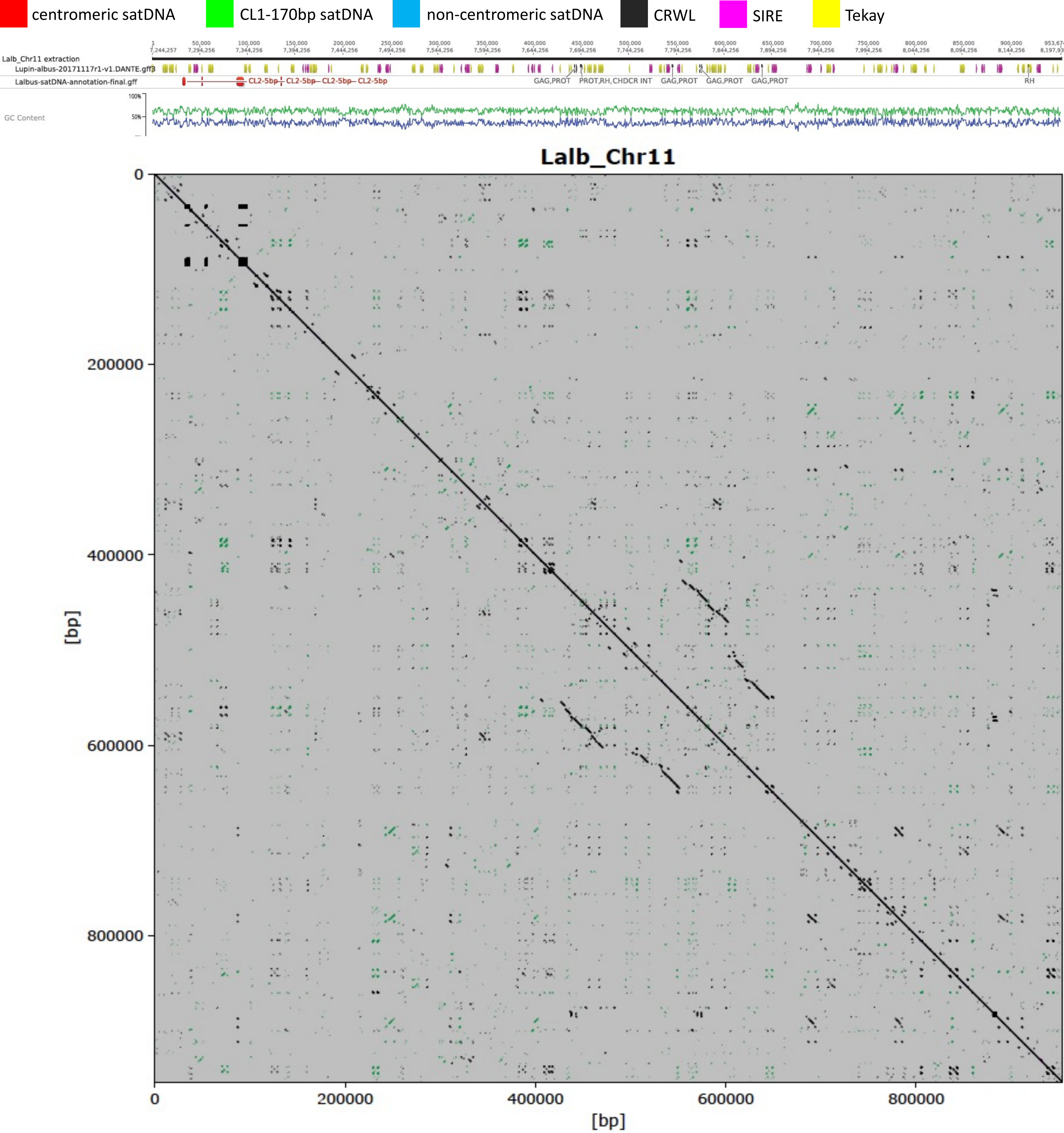

centromeric satDNA
CL1-170bp satDNA
non-centromeric satDNA
CRWL
SIRE
Tekay
Lalb_Chr11 extraction
Lupin-albus-20171117r1-v1.DANTE.gff3
Lalbus-satDNA-annotation-final.gff
CL2-5bp
GAG,PROT
PROT,RH,CHDCR INT
RH
GC Content
Lalb_Chr11
[bp]
0
200000
400000
600000
800000

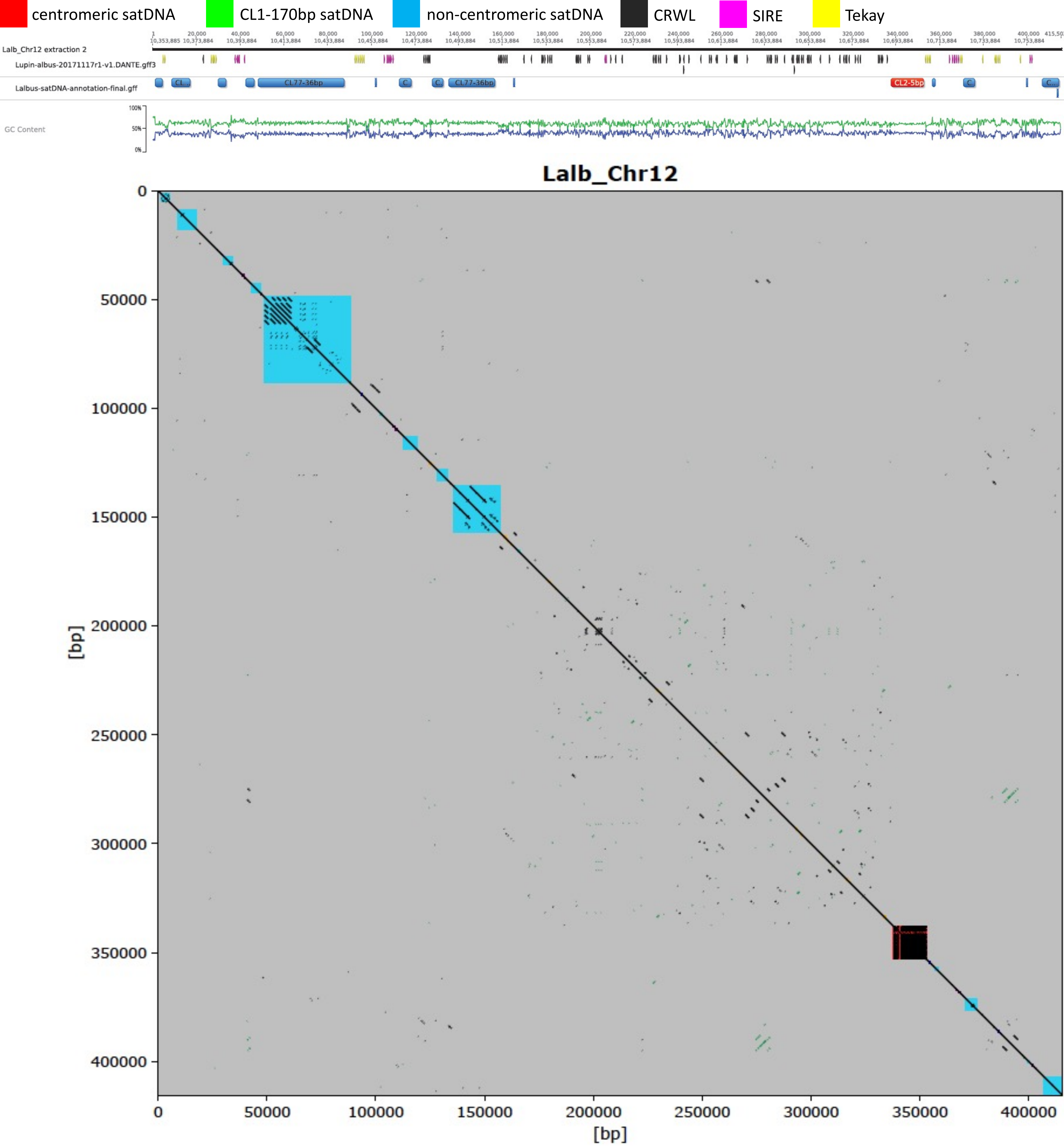

centromeric satDNA
CL1-170bp satDNA
non-centromeric satDNA
CRWL
SIRE
Tekay
Lalb_Chr12 extraction 2
Lupin-albus-20171117r1-v1.DANTE.gff3
Lalbus-satDNA-annotation-final.gff
CL77-36bp
CL77-36bp
CL2-5bp
GC Content
100%
50%
0%
Lalb_Chr12
[bp]
[bp]
0
50000
100000
150000
200000
250000
300000
350000
400000

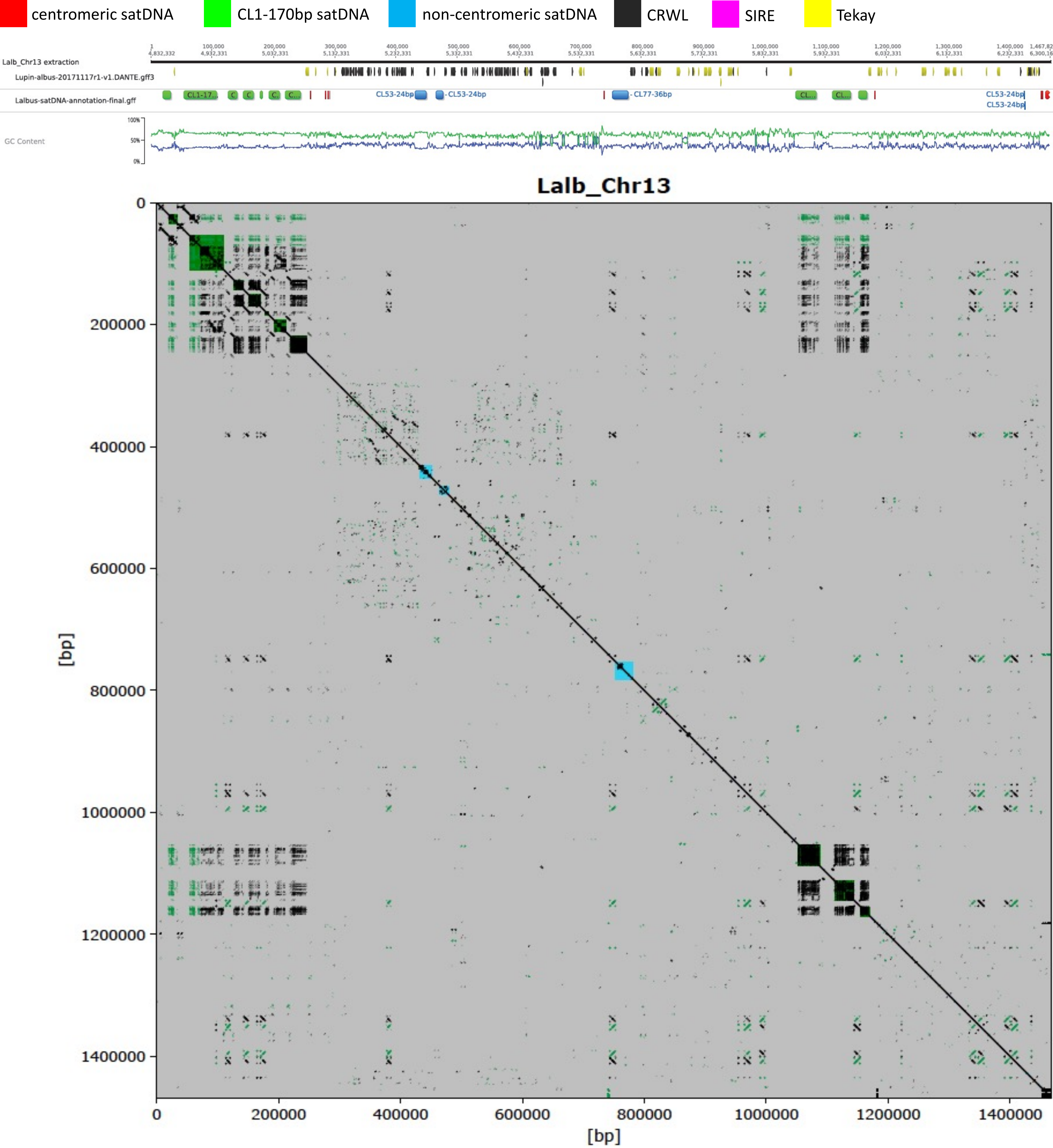
centromeric satDNA
CL1-170bp satDNA
non-centromeric satDNA
CRWL
SIRE
Tekay
Lalb_Chr13 extraction
Lupin-albus-20171117r1-v1.DANTE.gff3
Lalbus-satDNA-annotation-final.gff
CL53-24bp
CL77-36bp
GC Content
Lalb_Chr13
[bp]
[bp]

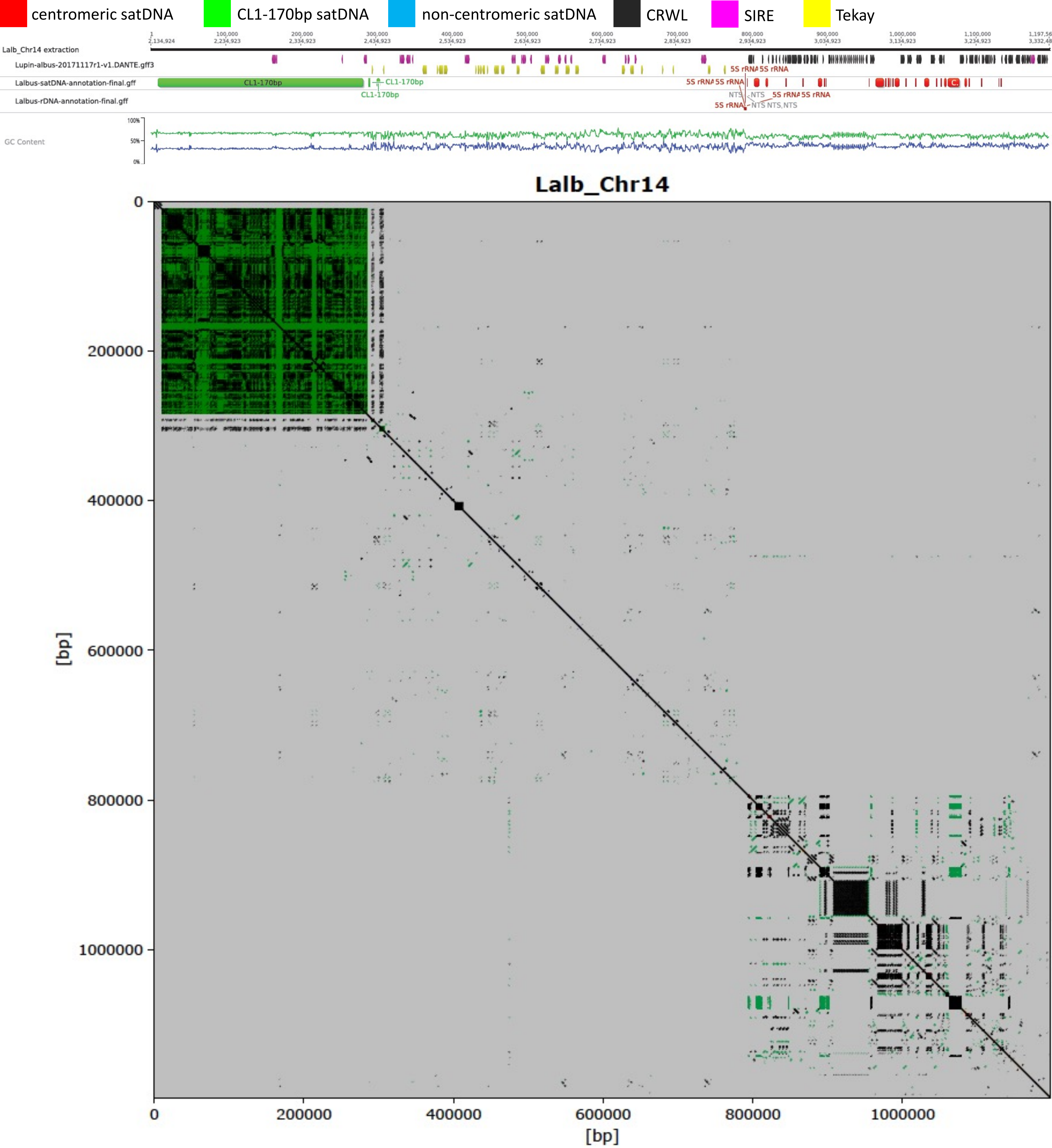

centromeric satDNA
CL1-170bp satDNA
non-centromeric satDNA
CRWL
SIRE
Tekay
Lalb_Chr14 extraction
Lupin-albus-20171117r1-v1.DANTE.gff3
Lalbus-satDNA-annotation-final.gff
Lalbus-rDNA-annotation-final.gff
CL1-170bp
5S rRNA
NTS
GC Content
100%
50%
0%
Lalb_Chr14
[bp]
0
200000
400000
600000
800000
1000000

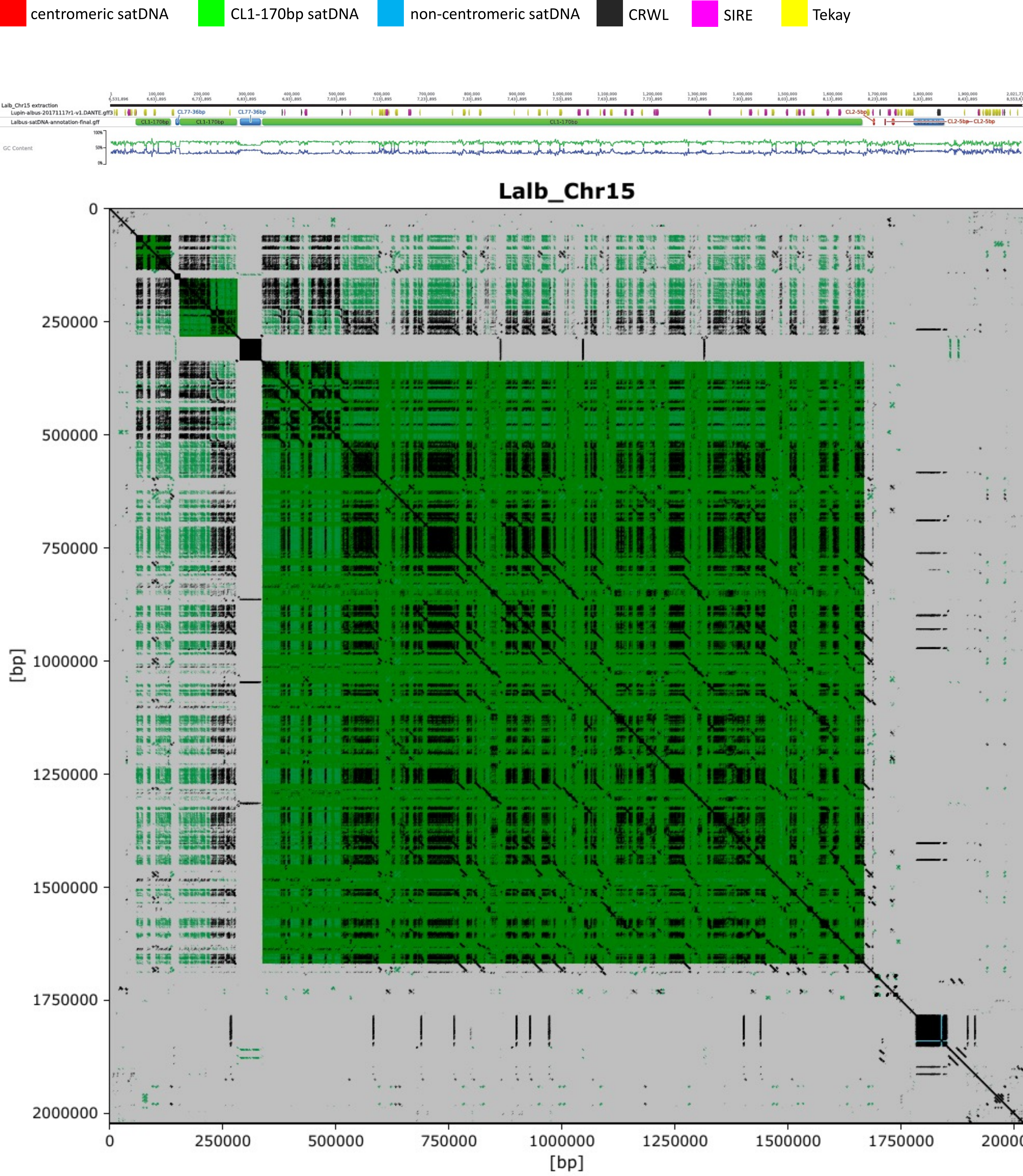

centromeric satDNA
CL1-170bp satDNA
non-centromeric satDNA
CRWL
SIRE
Tekay
Lalb_Chr15
[bp]
0
250000
500000
750000
1000000
1250000
1500000
1750000
2000000

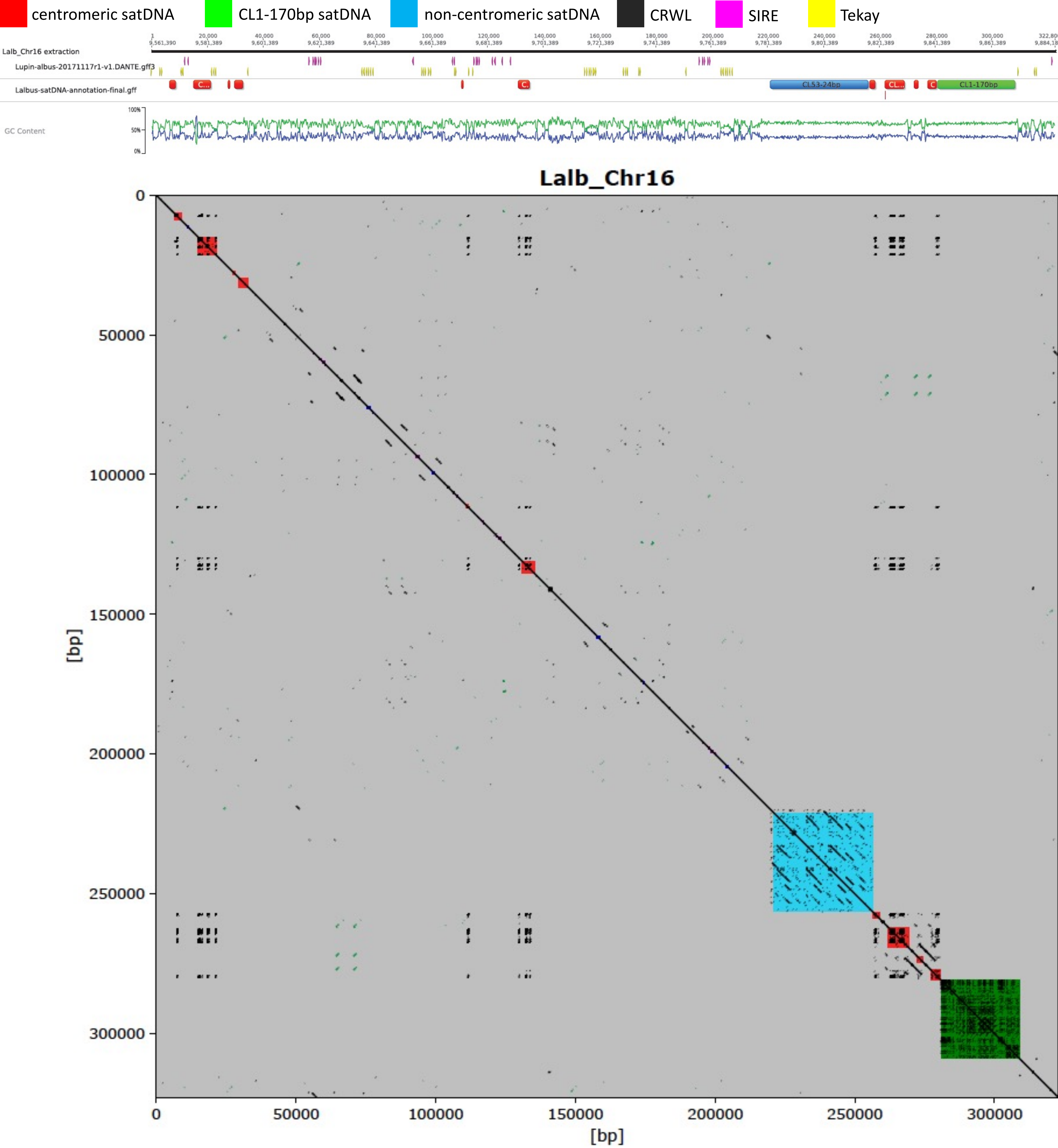

centromeric satDNA
CL1-170bp satDNA
non-centromeric satDNA
CRWL
SIRE
Tekay
Lalb_Chr16 extraction
Lupin-albus-20171117r1-v1.DANTE.gff3
Lalbus-satDNA-annotation-final.gff
CL53-24bp
CL1-170bp
GC Content
100%
50%
0%
Lalb_Chr16
[bp]
0
50000
100000
150000
200000
250000
300000

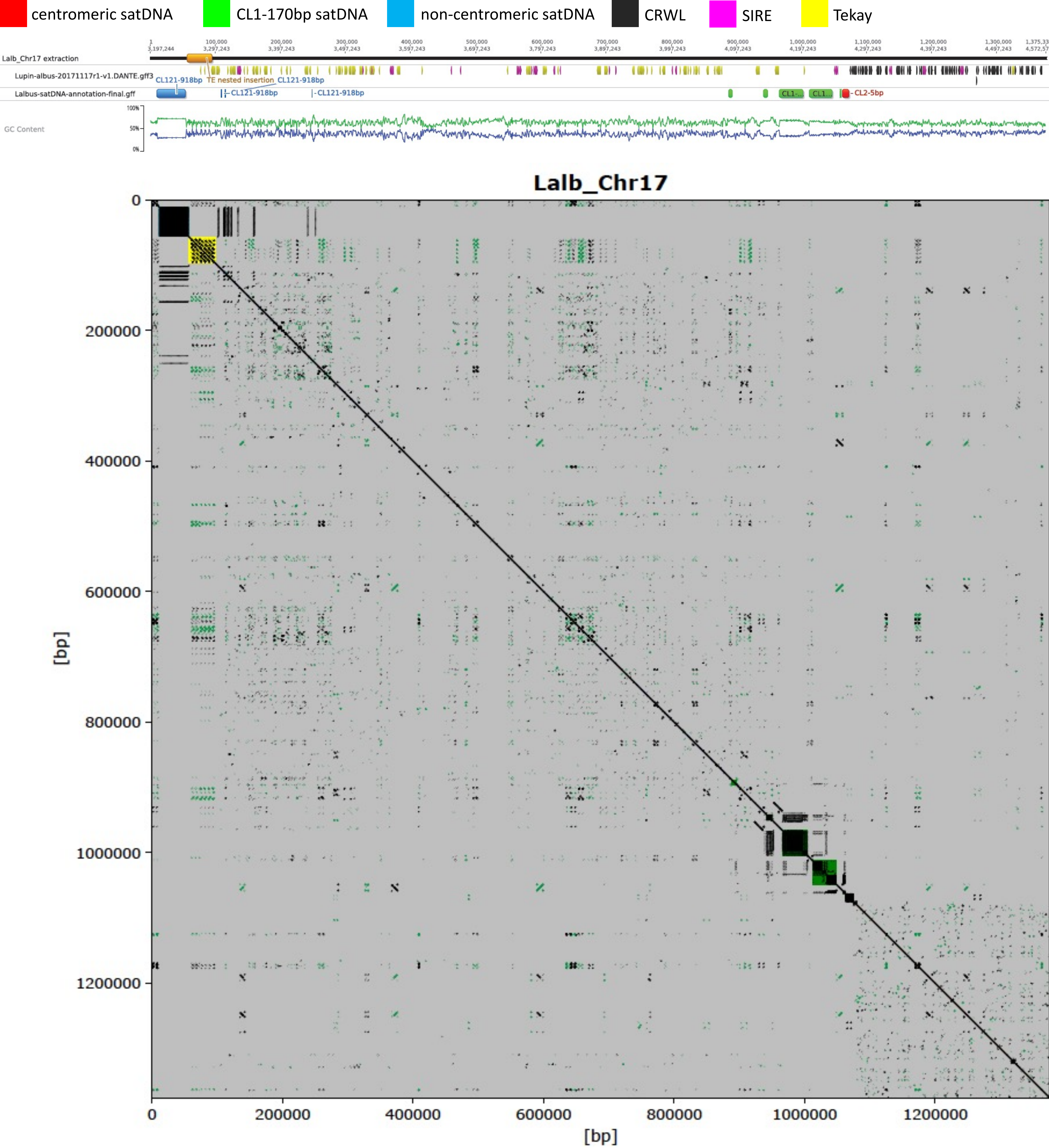

centromeric satDNA
CL1-170bp satDNA
non-centromeric satDNA
CRWL
SIRE
Tekay
Lalb_Chr17 extraction
Lupin-albus-20171117r1-v1.DANTE.gff3
Lalbus-satDNA-annotation-final.gff
CL121-918bp
TE nested insertion
CL2-5bp
GC Content
Lalb_Chr17
[bp]

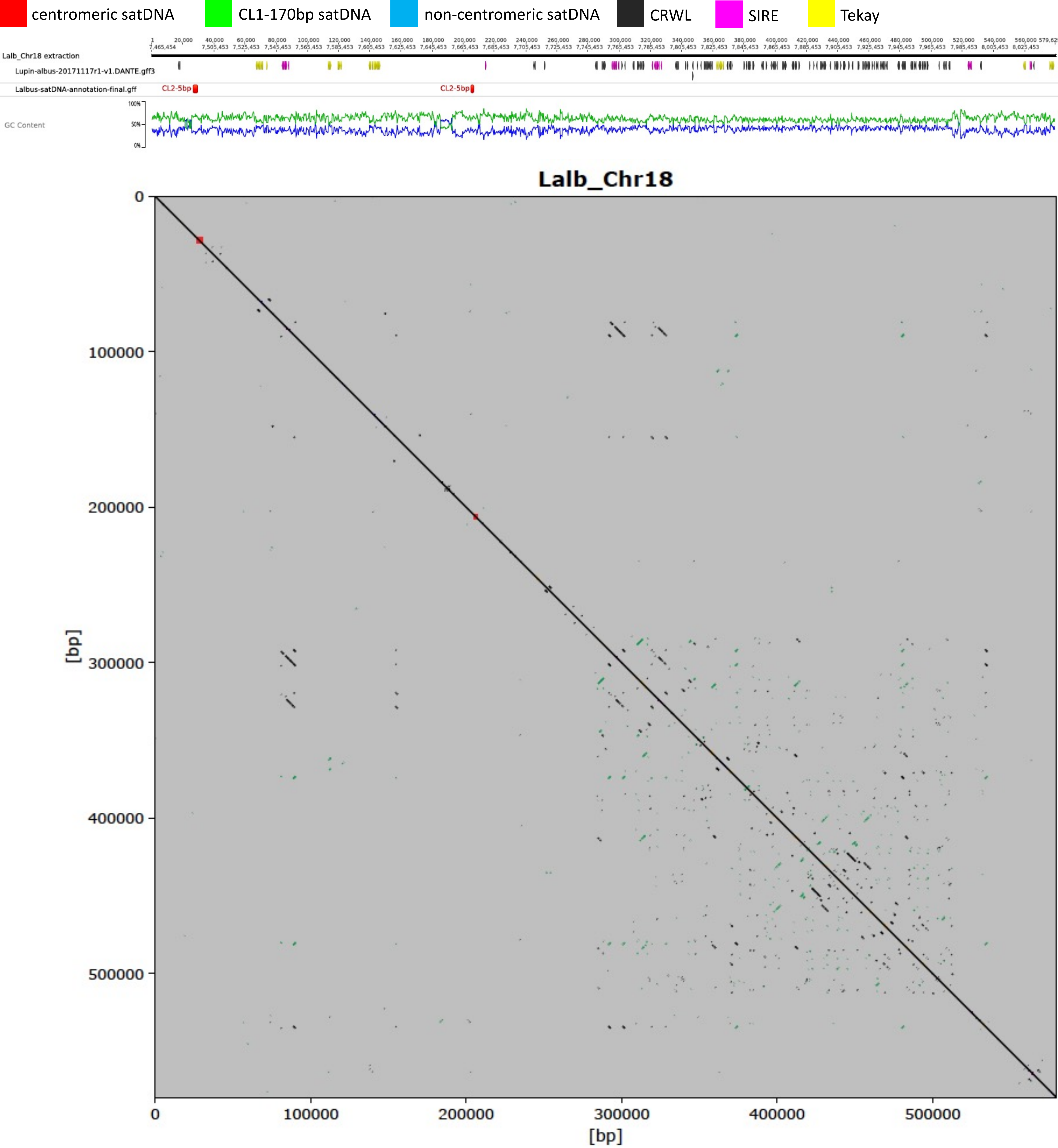

centromeric satDNA
CL1-170bp satDNA
non-centromeric satDNA
CRWL
SIRE
Tekay
Lalb_Chr18 extraction
Lupin-albus-20171117r1-v1.DANTE.gff3
Lalbus-satDNA-annotation-final.gff
CL2-5bp
CL2-5bp
GC Content
Lalb_Chr18
[bp]
[bp]

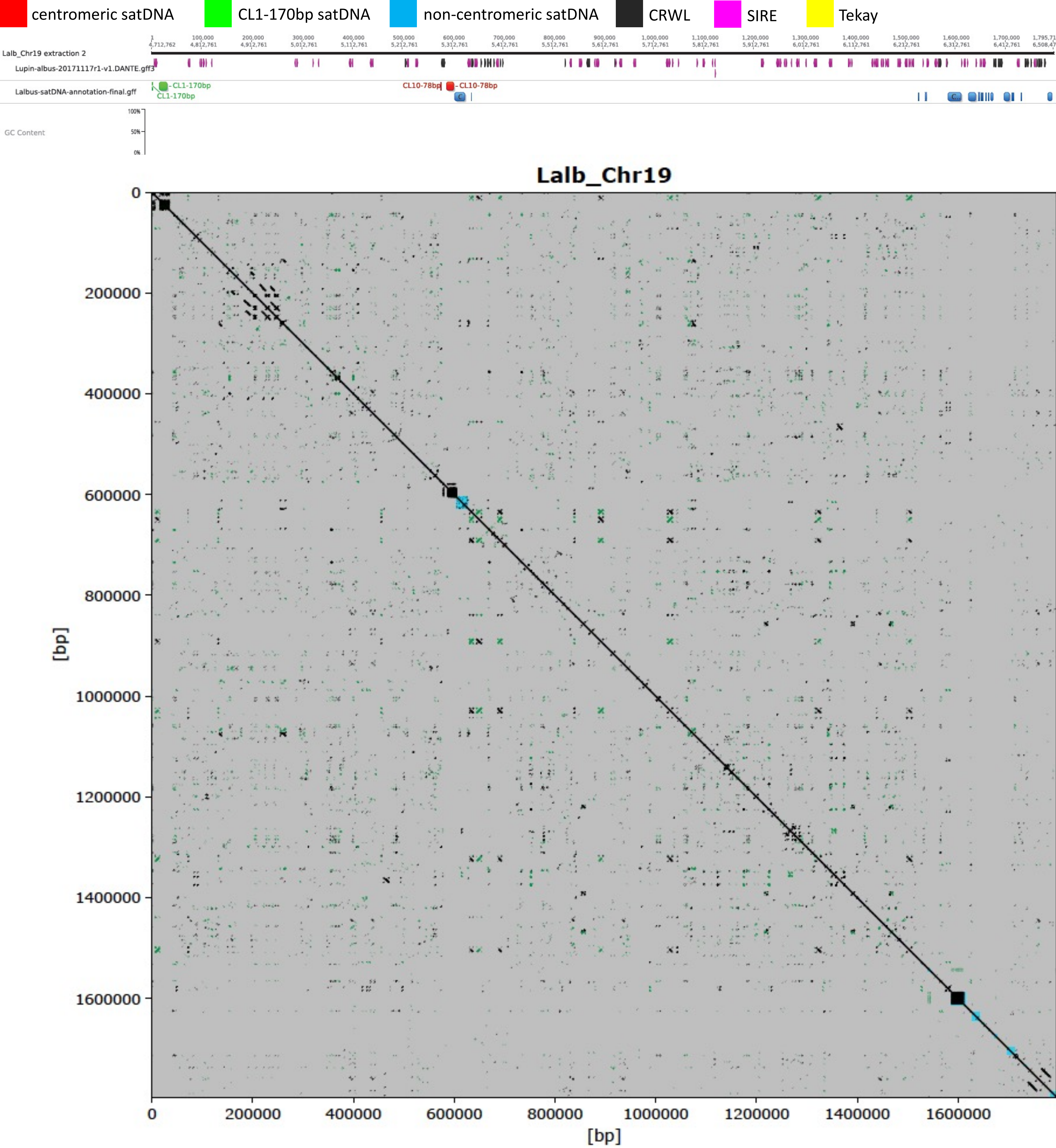
centromeric satDNA
CL1-170bp satDNA
non-centromeric satDNA
CRWL
SIRE
Tekay
Lalb_Chr19 extraction 2
Lupin-albus-20171117r1-v1.DANTE.gff3
Lalbus-satDNA-annotation-final.gff
CL1-170bp
CL10-78bp
GC Content
Lalb_Chr19
[bp]
0
200000
400000
600000
800000
1000000
1200000
1400000
1600000

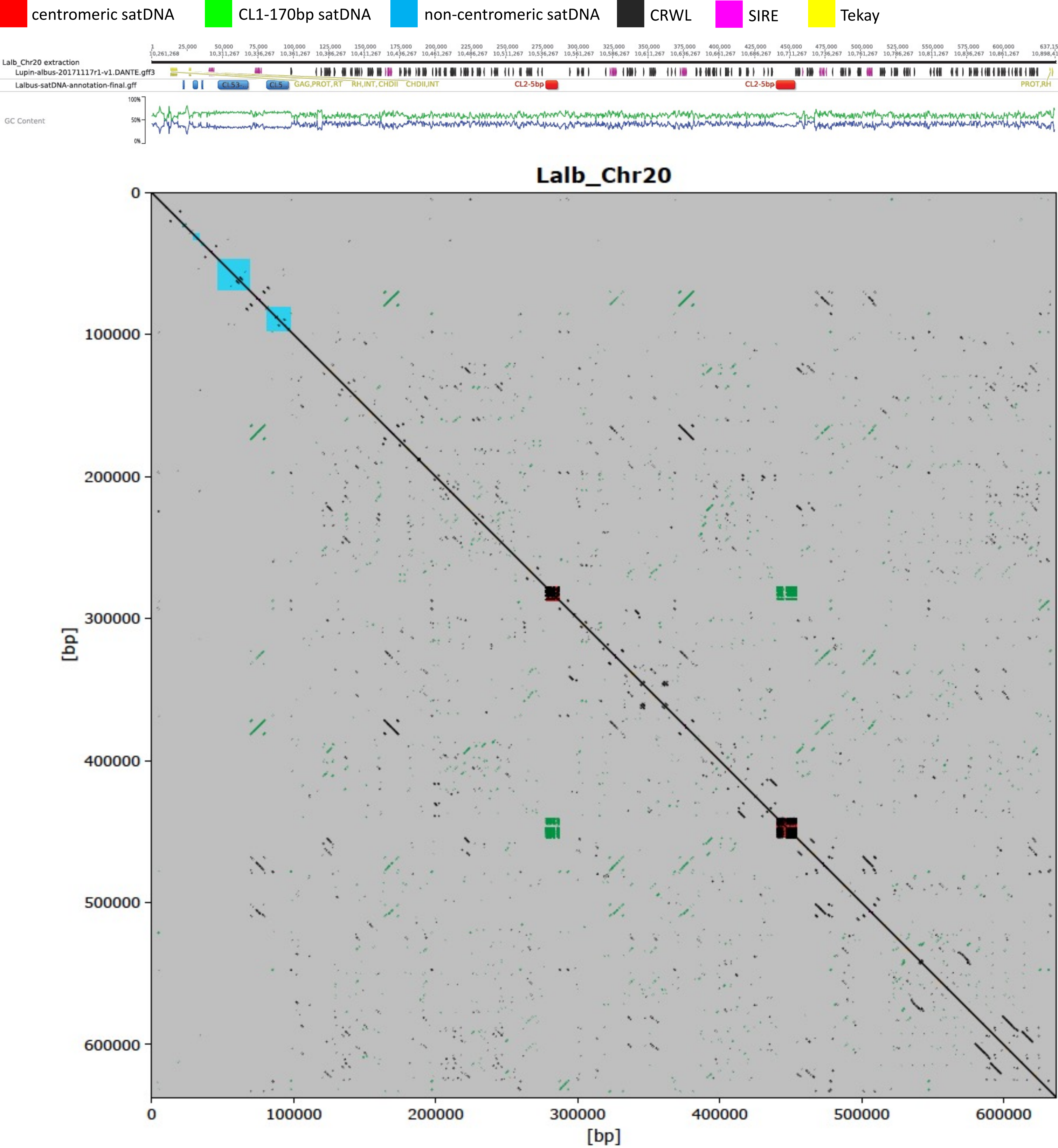

centromeric satDNA
CL1-170bp satDNA
non-centromeric satDNA
CRWL
SIRE
Tekay
Lalb_Chr20 extraction
Lupin-albus-20171117r1-v1.DANTE.gff3
Lalbus-satDNA-annotation-final.gff
GC Content
CL2-5bp
CL2-5bp
Lalb_Chr20
[bp]
[bp]
0
100000
200000
300000
400000
500000
600000

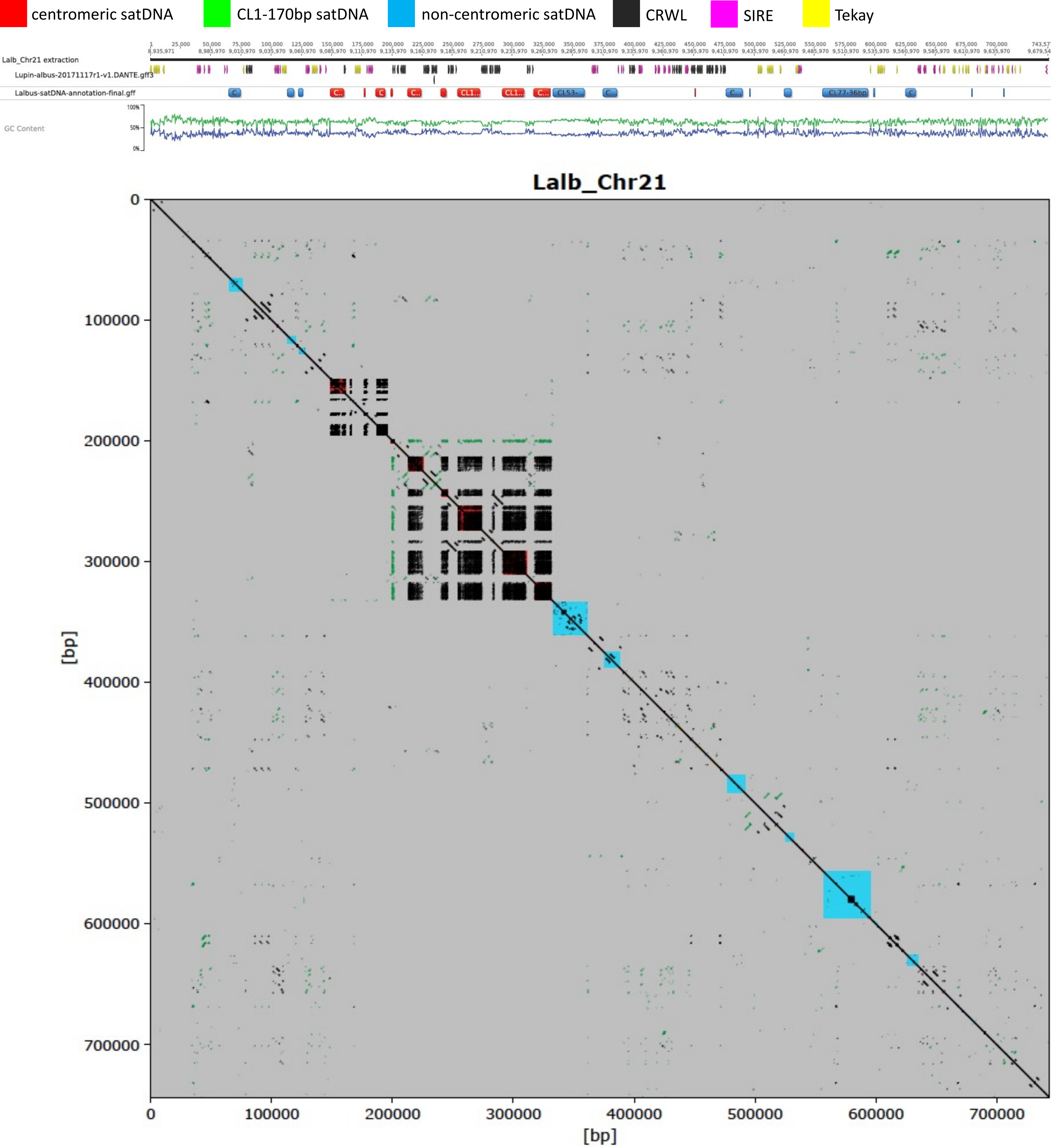

centromeric satDNA
CL1-170bp satDNA
non-centromeric satDNA
CRWL
SIRE
Tekay
Lalb_Chr21 extraction
Lupin-albus-20171117r1-v1.DANTE.gff3
Lalbus-satDNA-annotation-final.gff
GC Content
Lalb_Chr21
[bp]
[bp]

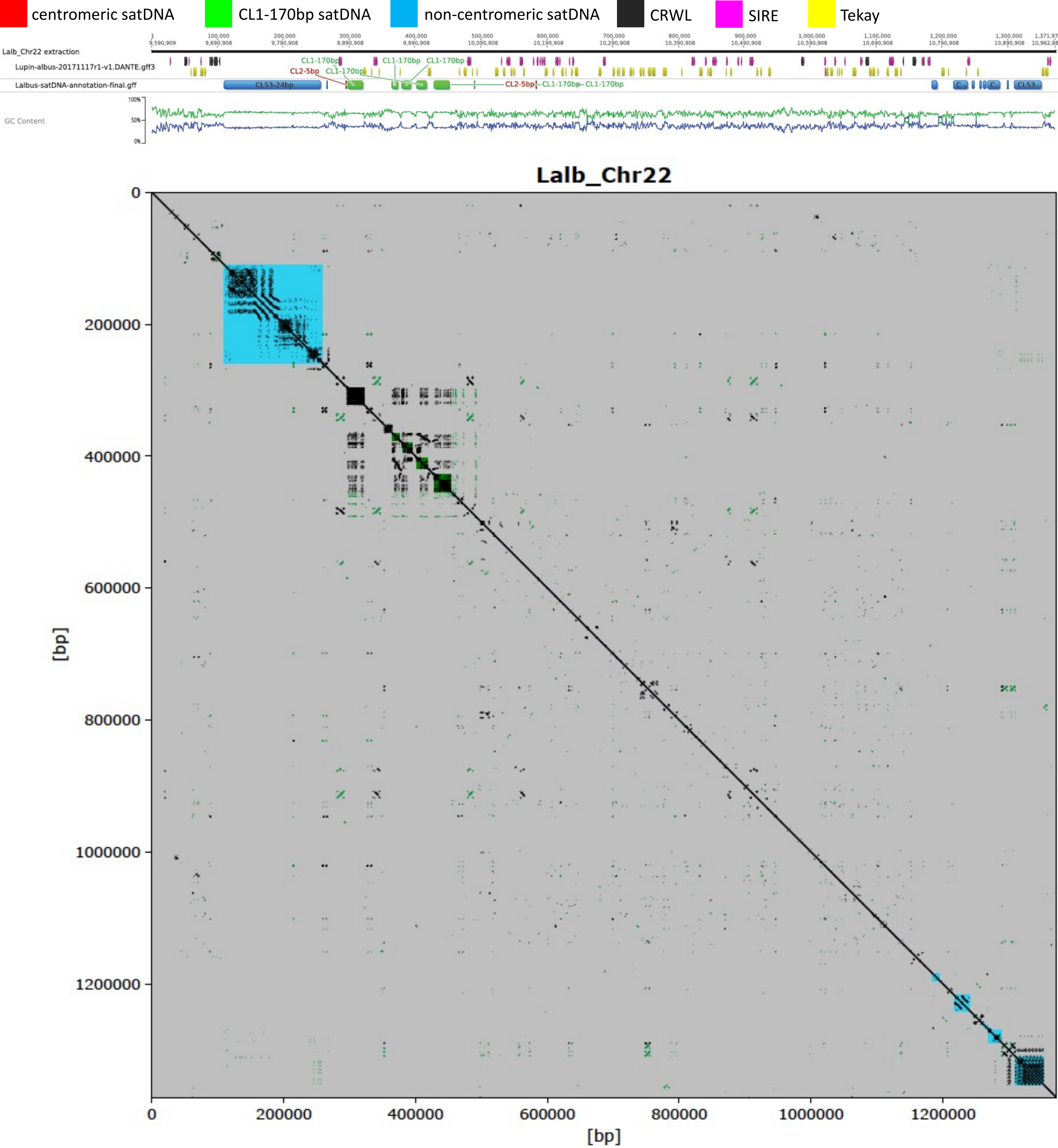

centromeric satDNA
CL1-170bp satDNA
non-centromeric satDNA
CRWL
SIRE
Tekay
Lalb_Chr22 extraction
Lupin-albus-20171117r1-v1.DANTE.gff3
Lalbus-satDNA-annotation-final.gff
CL53-24bp
CL1-170bp
CL2-5bp
GC Content
Lalb_Chr22
[bp]
[bp]

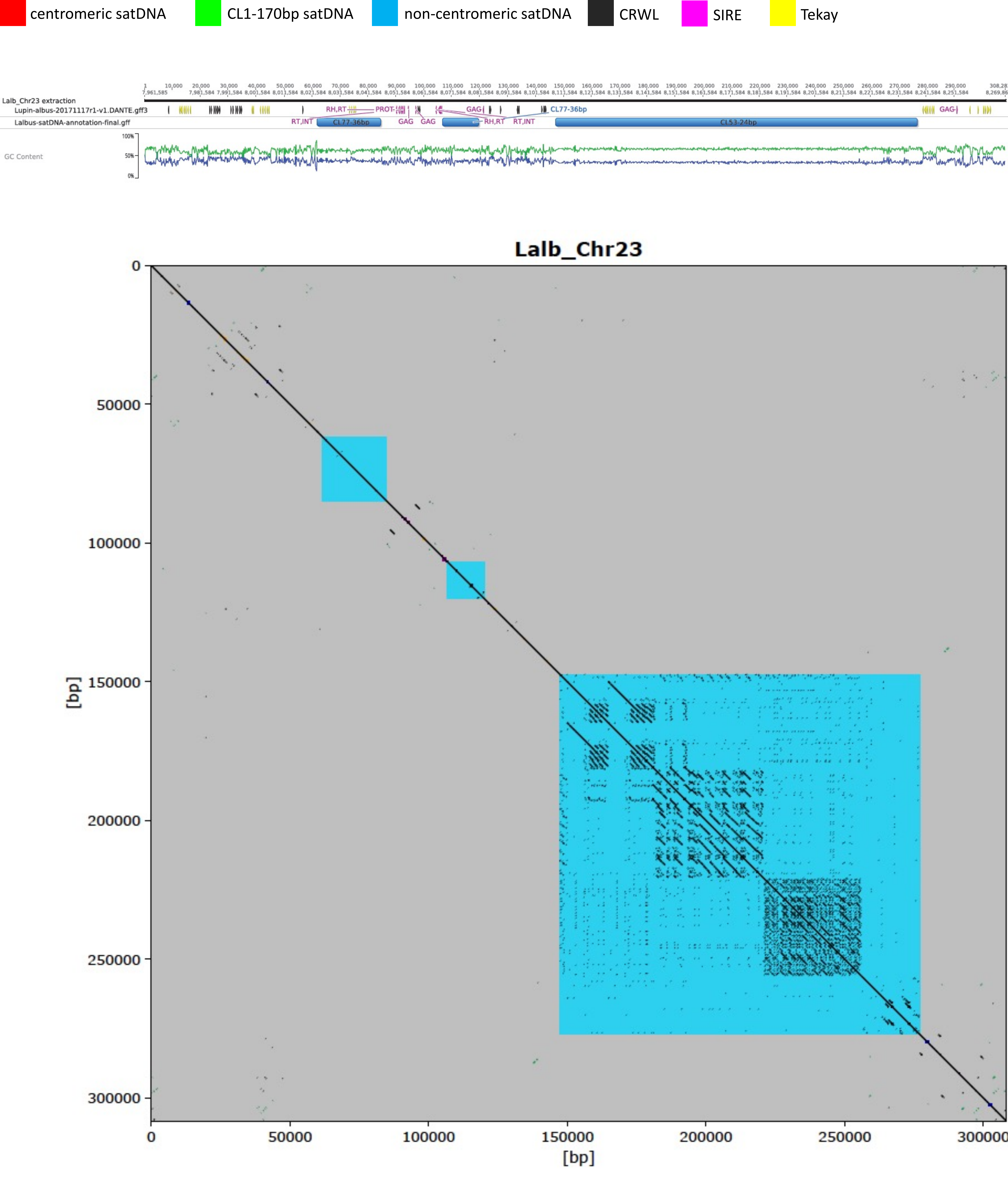

centromeric satDNA
CL1-170bp satDNA
non-centromeric satDNA
CRWL
SIRE
Tekay
Lalb_Chr23 extraction
Lupin-albus-20171117r1-v1.DANTE.gff3
Lalbus-satDNA-annotation-final.gff
RH,RT
PROT
GAG
RT,INT
CL77-36bp
CL53-24bp
GC Content
100%
50%
0%
Lalb_Chr23
[bp]
0
50000
100000
150000
200000
250000
300000

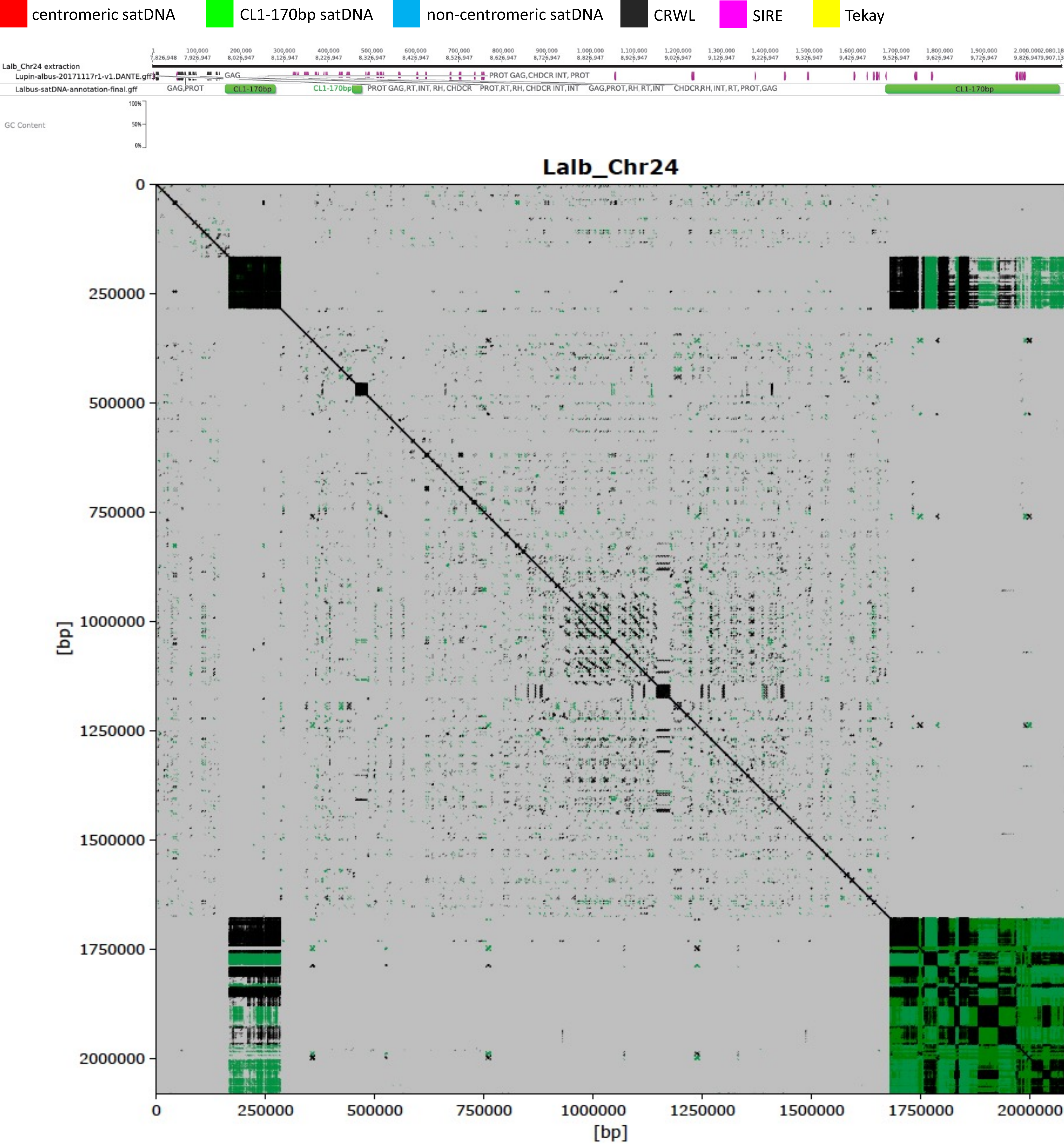

centromeric satDNA
CL1-170bp satDNA
non-centromeric satDNA
CRWL
SIRE
Tekay
Lalb_Chr24 extraction
Lupin-albus-20171117r1-v1.DANTE.gff3
Lalbus-satDNA-annotation-final.gff
GC Content
CL1-170bp
Lalb_Chr24
[bp]

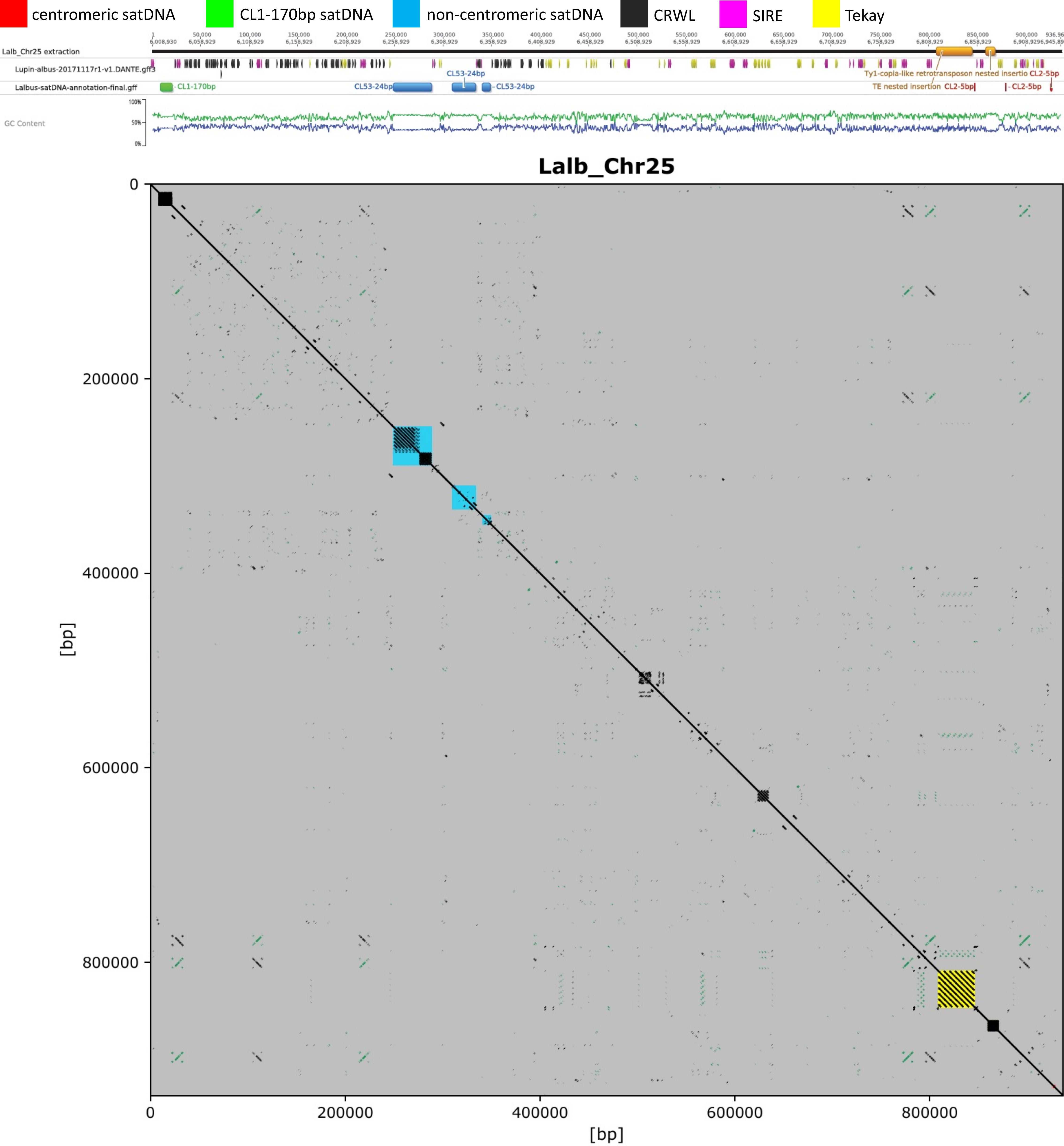

centromeric satDNA
CL1-170bp satDNA
non-centromeric satDNA
CRWL
SIRE
Tekay
Lalb_Chr25 extraction
Lupin-albus-20171117r1-v1.DANTE.gff3
Lalbus-satDNA-annotation-final.gff
CL1-170bp
CL53-24bp
CL53-24bp
CL53-24bp
Ty1-copia-like retrotransposon nested insertio
CL2-5bp
TE nested insertion
CL2-5bp
CL2-5bp
GC Content
Lalb_Chr25
[bp]
[bp]
